# D-stem mutation in an essential tRNA increases translation speed at the cost of fidelity

**DOI:** 10.1371/journal.pgen.1011569

**Published:** 2025-02-04

**Authors:** Madison N. Schrock, Krishna Parsawar, Kelly T. Hughes, Fabienne F. V. Chevance

**Affiliations:** 1 School of Biological Sciences, University of Utah, Salt Lake City, Utah, United States of America; 2 Analytical and Biological Mass Spectrometry Core, University of Arizona, Tucson, Arizona, United States of America; University of Wisconsin-Madison, UNITED STATES OF AMERICA

## Abstract

The efficiency with which aminoacyl-tRNA and GTP-bound translation elongation factor EF-Tu recognizes the A-site codon of the ribosome is dependent on codons and tRNA species present in the polypeptide (P) and exit (E) codon sites. To understand how codon context affects the efficiency of codon recognition by tRNA-bound EF-Tu, a genetic system was developed to select for fast translation through slow-translating codon combinations. Selection for fast translation through the slow-translated UCA-UAC pair, flanked by histidine codons, resulted in the isolation of an A25G base substitution mutant in the D-stem of an essential tRNA LeuZ, which recognizes the UUA and UUG leucine codons. The LeuZ(A25G) substitution allowed for faster translation through all codon pairs tested that included the UCA codon. Insertion of leucine at the UCA serine codon was enhanced in the presence of LeuZ(A25G) tRNA. This work, taken in context with the Hirsh UGA nonsense suppressor G24A mutation in TrpT tRNA, provides genetic evidence that the post-GTP hydrolysis proofreading step by elongation factor Tu may be controlled by structural interactions in the hinge region of tRNA species. Our results support a model in which the tRNA bending component of the accommodation step in mRNA translation allows EF Tu time to enhance its ability to differentiate tRNA interactions between cognate and near-cognate mRNA codons.

## Author summary

The translation of mRNA into protein by the ribosome is one of the most fundamental processes in Biology. GTP-bound elongation factor EF-Tu brings charged aminoacyl-tRNAs to the A-site in the ribosome decoding center to determine if the anti-codon of the tRNA is correct for a given codon being read. Differentiating proper cognate codon recognition from near-cognate codons is coupled to GTP hydrolysis and a conformational bending of the tRNA in the hinge region of the tRNA structure. We show that a tRNA mutant with a single base substitution in the hinge region of the tRNA allows for enhanced near-cognate codon recognition and an increase in the speed of codon translation. This result suggests an evolutionary trade-off between speed of codon recognition by the translation machinery and fidelity of the translation process.

## Introduction

**Fig 1:**
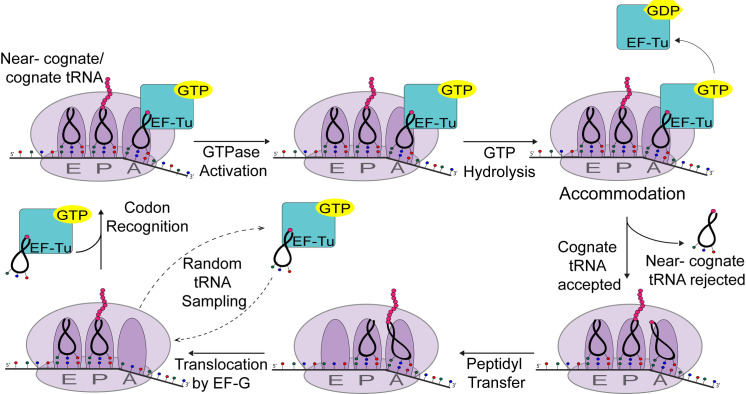
Overview of translation elongation. tRNA’s are randomly brought into the A-site by GTP-bound elongation factor Tu (EF-Tu). If a non-cognate tRNA is brought in, it is immediately rejected. If a near-cognate or cognate tRNA is brought in for potential recognition, the ribosomal subunits start assessing full complementarity between the anticodon and codon, allowing for GTPase activation. The large ribosomal subunit will change conformation causing the tRNA in the A-site to bend, allowing EF-Tu bound to GTP to interact with the catalytic residues responsible for GTP hydrolysis. As the tRNA in the A-site relaxes, EF-Tu dissociates from the ribosome and accommodation of the cognate tRNA occurs. Near- cognate tRNA’s are rejected prior to accommodation. Accommodation of the tRNA into the A-site positions the 3’ CCA end of the tRNA for peptidyl transfer. Elongation factor **G** (EF-G) promotes peptide bond formation and translocation of the ribosome.

DNA is transcribed by RNA polymerase into messenger RNA (mRNA), which is then translated into protein by the ribosome. In prokaryotes, the ribosome is composed of 50s (large) and 30s (small) subunits. During translation elongation, charged, aminoacyl transfer RNAs (tRNAs) interact with mRNA codon positions within the ribosome decoding center: the aminoacyl (A-), peptidyl (P-), or exit (E-) sites. Translation elongation involves four principal steps: 1) recognition of the incoming aminoacyl-tRNA, 2) accommodation of the aminoacyl-tRNA into the A-site, 3) peptide bond formation between adjacent amino acid residues, and 4) translocation of the ribosome down the mRNA (Fig 1).

Selection of the correct aminoacyl-tRNA whose anticodon base pairs with the mRNA codon in the A-site of the ribosome during elongation is essential for translation fidelity. GTP-bound elongation factor-Tu (EF-Tu) randomly brings aminoacylated tRNAs to the A-site for potential recognition at a rate of about 1,000 times per second [1–5]. The 30s ribosomal subunit then assesses complementarity of the aminoacyl-tRNAs anticodon and the mRNA codon in the ribosomal A-site [6,7]. When the two bases of the codon (usually the first two bases) are complementary to the tRNA’s anticodon, the aminoacyl-tRNA remains in the A-site of the ribosome to assess pairing of all three bases. This is especially important at the wobble base, which has less strict base-pairing rules [8,9]. If a near-cognate or non-cognate aminoacyl-tRNA is located in the A-site, the ribosome senses an incorrect match and the aminoacyl-tRNA is rejected [10]. As the anticodon/codon complementary is being assessed, the ribosome undergoes a conformational change, which induces a bending in the tRNA. This in turn allows the GTP-binding domain of EF-Tu to interact with the L7/L12 stalk on the 50s ribosomal subunit and catalyze the hydrolysis of GTP [11–20]. Following GTP hydrolysis, the aminoacyl-tRNA is accommodated into the A-site [18,21], EF-Tu and GDP dissociate from the ribosome, and elongation factor-G facilitates translocation of the ribosome by three bases [22].

Translation is fine-tuned to maximize fitness of an organism by balancing translation speed with accuracy of amino acid incorporation into the growing polypeptide chain. Failure of translation to proceed at an appropriate rate can result in changes in mRNA stability, lowered protein expression, and even the synthesis of non-functional proteins [23–26]. There are two fidelity checks that an aminoacyl-tRNA in the A-site must undergo to maintain accuracy of translation. The first is anticodon-codon complementarity [9,27]. The second is sufficient energy supplied following a conformational change in the ribosome structure that results in a bending of the tRNA that is coupled to GTP hydrolysis [18,27,28]. The rate at which the correct aminoacyl-tRNA is incorporated into the A-site depends on its relative abundance in the cellular tRNA pool, which controls translation speed [29,30]. Other factors that contribute to translation efficiency include codon context and tRNA modifications [31–33]. Codon context can influence the speed of elongation to ensure appropriate expression of individual genes [34]. Codon context effects on translation occur when neighboring codons and tRNAs in the E-site and P-site influence A-site aminoacyl-tRNA recognition [31,33].

**Fig 2:**
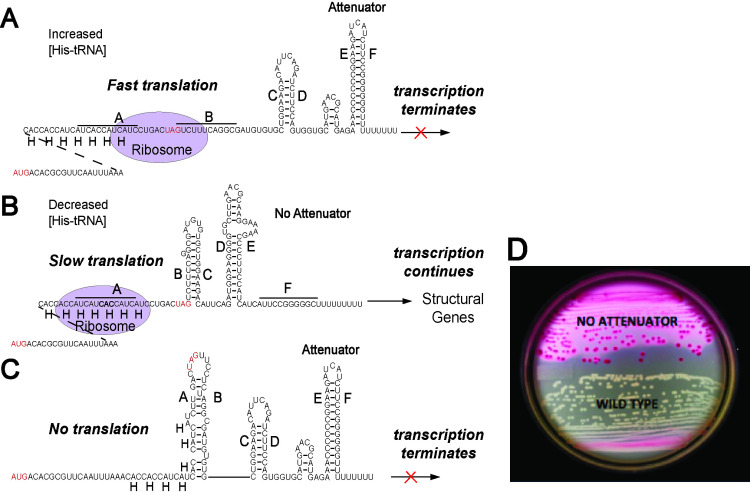
The *his* operon leader peptide control region. **(A)** The *his* operon leader peptide region contains a 16 amino acid open reading frame with seven consecutive histidine (His) codons and RNA sequences that can form alternate secondary structures. At high levels of charged histidyl-tRNA, fast translation through the seven histidine codons results in the formation of a strong Rho-independent stem-loop structure (E-F) that attenuates further transcription by RNA polymerase into the downstream structural genes encoding the histidine biosynthetic enzymes. **(B)** Low levels of charged histidyl-tRNA results in stalled translation through the seven histidine codons of the leader peptide coding region, which results in alternative RNA secondary structures that lack the attenuator. This allows RNA polymerase to continue transcription through the *his* operon structural genes. **(C)** When transcription of the *his* leader peptide region is not coupled to translation, the default RNA secondary structures include the formation of the strong Rho-independent terminator. **(D)** Fusion of the *lac* operon to the *hisD* structural gene (*his****D:****:*Mud-*lac*) allows for a simple phenotypic determination on MacConkey lactose (Mac-Lac) indicator medium where attenuator formation in the *his* leader region results in low levels of *his-lac* transcription and a Lac^-^ or white colony phenotype. Loss of attenuation in the his leader region results in high levels of *his-lac* transcription and a Lac^+^ or red colony phenotype and the precipitation of bile salts present in the medium.

To gain a better understanding of the effects of codon context on translation, we report here a genetic selection to isolate fast translation mutants for codon pairs that are translated slowly. It utilizes the *Salmonella* histidine (*his*) biosynthetic operon control region, which acts as a translation speedometer through His codons. The region between the *his* operon promoter and the biosynthetic structural genes includes a small leader peptide gene sixteen codons long that includes seven consecutive His codons (Fig 2A–C). The leader peptide gene is followed by an RNA stem-loop Rho-independent transcriptional attenuator. Formation of this attenuator stem-loop prevents the continuation of RNA polymerase transcription into the *his* structural genes [35–37]. An alternative RNA secondary structure can also form that disrupts the attenuator stem-loop and allows transcription of the *his* operon structural genes. Attenuator formation depends on the speed that ribosomes move through the consecutive His codons. This translation speedometer allows the bacterium to assay the cellular concentration of charged histidyl-tRNA and produce the required amount of His biosynthetic enzymes. Transcription into the *his* structural genes demands stalling of the ribosome in the consecutive His codons. If the level of charged histidyl-tRNA is high, ribosome stalling does not occur, the ribosome continues behind RNA polymerase to the stop codon of the leader peptide sequence. This results in attenuator formation, removal of RNA polymerase from the DNA and no further *his* operon transcription. Importantly, this translation speedometer assay is independent of protein and mRNA stability [38]. We have shown that the *his* leader peptide system can be used to measure translation rates through individual codons or through multiple codon combinations [39]. Measurement of β-galactosidase expression of a *lac* operon reporter fused to the *hisD* gene allows for a quantitative readout through measurement of β-galactosidase activity. These assays provide an indirect measurement of translation speed through any codon that is placed in the run of consecutive His codons in the leader peptide gene. Furthermore, the use of MacConkey-lactose (Mac-Lac) indicator medium allows for a simple visual qualitative readout as colonies vary in color from white (Lac^-^) to pink to red (Lac^+^) with increasing β-galactosidase (Fig 2D). The highest levels of β-galactosidase activity result in the precipitation of bile salts present in the indicator medium.

The *his* leader peptide system has been used to determine the effect of codon context on translation speed through codon pairs [36,37,39]. This work revealed the importance of codon orientation (context) on the efficiency of translation through codon pairs [39]. For example, translation through the UAC-UCA (Tyr-Ser) codon pair was fast in the *his* leader peptide system while in the reverse order, translation of UCA-UAC was slow, resembling what was observed in assays with stop codons. In the present study, we modified the translation speedometer assay in a way that allows selection for faster translation through translation-slow codons. A selection for faster translation through the translation-slow UCA-UAC codon pair resulted in the isolation of a surprising mutation in the D-stem of tRNA^LeuZ^ (there are no leucine codons present in the *his* leader peptide sequence). Further analysis revealed that this mutant tRNA^LeuZ^ causes increased translation speeds in the *his* leader through many different codon pairs where UCA was one of the two codons in the pair being assayed. We describe the isolation of this tRNA mutation and its properties and discuss its implications for understanding control of translation speed and fidelity.

## Results

### The slow-translating phenotype of a UCA-UAC codon pair at positions His4 and His5 of the *his* leader peptide region is suppressed in *cis* by changing adjacent codons

**Fig 3:**
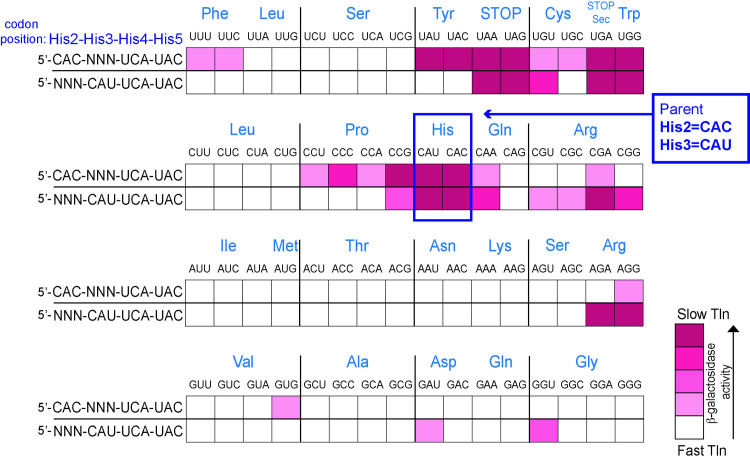
Effect of adjacent codons on the slow translation phenotype through the UCA-UAC codon pair placed at the His4-His5 codon positions in the *his* leader peptide. The presence of UCA-UAC at the His4-His5 positions in the leader peptide sequence resulted in a translation-slow phenotype similar to what was observed with a stop codon placed at either his4 or His5 [39]. The effect of codons at positions His2 and His3 on the slow translation phenotype of UCA-UAC at His4-His5 was determined. The His2 and His3 codons were changed to all 64 codons and the effect on translation through the *his* leader peptide was assayed on Mac-Lac indicator medium using a *his-lac* reporter (*hisD9953*::Mud-*lac*) to measure *his* operon transcription levels. The Mac-Lac plate phenotypes are shown as ranging from white (Lac^-^) to dark red (Lac^+^). Intermediate Mac-Lac plate phenotypes are indicated by the gradation of darkening pink colors. The Mac-Lac color phenotypes represent an indirect readout of the speed of translation through the *his* leader peptide region.

In the *his* leader peptide translation speedometer system, apparent translation rates can be measured using a *hisD::*Mud-*lac* transcriptional reporter. Visualization of colony color intensity – due to the expression of the *lac* operon – on lactose-utilization indicator plates can be used to estimate speed of translation through the *his* leader gene, and expression can be quantified by measuring β-galactosidase (β-gal) activity. On MacConkey-lactose (Mac-Lac) indicator plates, fermentation of lactose lowers the pH of the colony and depending on the level of *lac* expression, colony color varies from a white (Lac^-^) to dark red (Lac^+^). As mentioned above, a UCA-UAC codon pair placed at the His4-His5 positions in the *his* leader peptide region exhibited a translation-slow phenotype yet the reverse orientation with UAC-UCA at His4-His5 was translated fast [39]. We tested for potential *cis*-acting effects on translation through the UCA-UAC slow-translating pair by changing the upstream codons at positions His2 and His3. All 64 codons were incorporated at either the His2 or His3 codon positions upstream of UCA-UAC at His4-His5 in the *his* leader peptide sequence and their effect on the expression of a *hisD-lac* operon reporter was determined on lactose indicator medium. Mac-Lac indicator plates were used to provide a qualitative screen for suppression of the translation-slow phenotype. The variation in degrees of translation stalling translates to variation of *hisD-lac* operon expression, which was visualized on lactose indicator medium. Fast translation through the *his* leader sequence occurs with all seven His codons present and results in a Mac-Lac white Lac^-^ colony phenotype in this assay (Fig 2D). The translation-stalled phenotype of UCA-UAC codons at His4-His5 of the leader peptide exhibits a strong Lac^+^ phenotype (red colony with bile salts precipitation). With the UCA-UAC codons at His4-His5 in our assay, the addition of substitutions at His3 resulted in only four coding codons, UAU (Tyr), UAC (Tyr), UGG (Trp) and CCG (Pro), that retained the translation-slow phenotype of UCA-UAC at His4-His5 (Fig 3). The remaining fifty-seven coding codons introduced at His3 resulted in faster translation phenotypes (Fig 3). Similarly, for substitutions at His2, only four coding codons, UGG (Trp), CGA (Arg), AGA (Arg) and AGG (Arg), retained the translation-slow phenotype of UCA-UAC at His4-His5. Most substitutions at either His2 or His3 resulted in a faster-translation Lac^-^ phenotype as compared to the translation-stalled Lac^+^ parent strain phenotype (Fig 3). It is worth noting that the slow translation phenotype of the UCA-UAC at His4-His5 is in the context of flanking histidine codons. These results are consistent with earlier observations suggesting that codon context can have a profound effect on the efficiency of mRNA translation [34].

### A *hisD-sacB* reporter provides a positive selection against slow translation speed in the *his* leader peptide translation speedometer system

**Fig 4:**
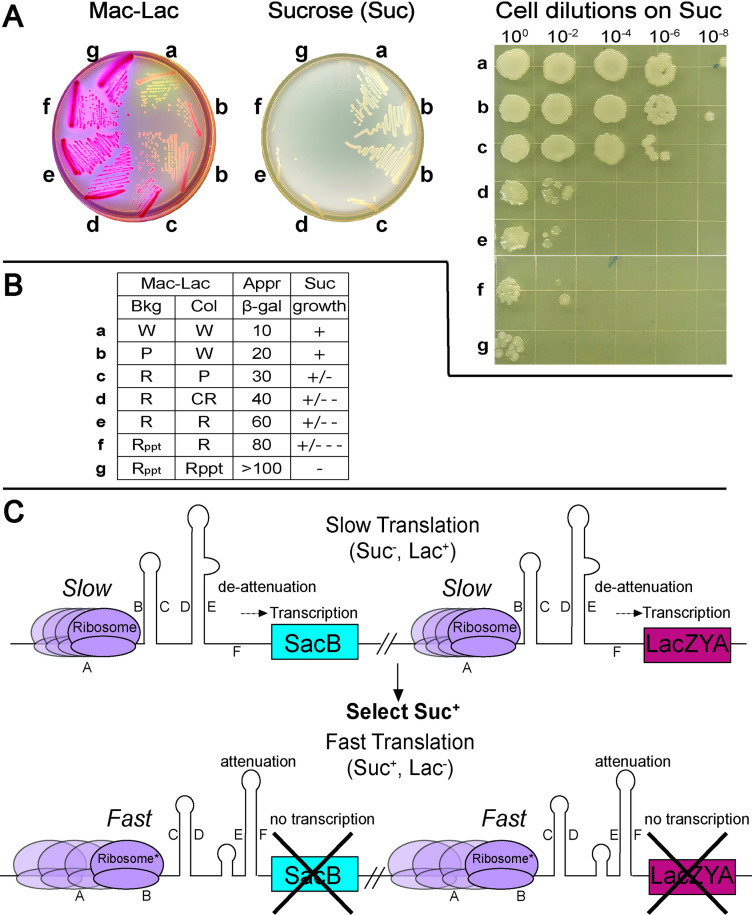
Use of *sacB* and *lac* operon reporters to select and screen for mutants resulting in faster translation in the *his* leader peptide region. **(A)** Growth phenotypes on MacConkey lactose (Mac-Lac) and sucrose-containing indicator plates for strains containing either *lac* operon or *sacB* reporters expressed from the *his* operon control region that have different speeds of translation through the *his* leader peptide region. A fast-translation speed (labeled A) results in a Lac^-^, white colony phenotype and growth on sucrose-containing media for *his*-*lac* or *his*-*sacB* reporter constructs, respectively. A slow-translation speed (labeled G) results in a Lac^+^ with precipitation of bile salts red colony phenotype and no growth on sucrose-containing media for *his*-*lac* or *his*-*sacB* reporter constructs, respectively. Intermediate translation speeds are labeled B through F. Classification of lactose phenotype are indicated by a letter from A-G. Δ*his****D*:**:*sacB,* in response to different translation rates results in growth or no growth on 6% sucrose media, +: robust growth, +/-: smaller single colonies and mucoid phenotype, +/--: no single colonies and mucoid phenotype, and -: no growth. Phenotypes for Mac-Lac were determined using strains TH2141, TH28870, TH28871, TH28872, TH28873, TH28874, TH28875. A 6% sucrose plate with the range of life/death seen with A-G. Phenotypes for sucrose growth phenotypes were determined using strains TH27455, TH28679, TH28680, TH28781, TH28782, TH28783, TH28784. **(B)** The approximate β-galactosidase (β-gal) activities and growth phenotypes on sucrose (Suc) medium associated with the different *his* operon expression levels (A through G) are indicated. (W = white, **P** = pink, **R** = red, CR = red colony center, R_PPT_ = red colony with precipitated bile salts). **(C)** A strain that is diploid for the *his* biosynthetic operon was constructed to select for mutants resulting in a faster-translation speed through the *his* leader peptide region. One of the duplicated *his* operon regions contains a replacement of the *hisD* structural gene with the *sacB* coding sequence; the second *his* operon contains a *lac* operon fusion to the *hisD* structural gene. The resulting diploid strain was used to isolate mutants that were simultaneously defective in the expression of both *his* operons so that both the *sacB* and *lac* operon reporters were not expressed. Stalled translation through the *his* leader peptide region results in a G phenotype (Suc^-^ Lac^+^). Fast translation is selected for on 6% sucrose plates, and then screened on MacConkey- lactose indicator plates for mutants with a Suc^+^ Lac^-^ phenotype. In this study TH27508 was used to isolate fast-translation mutants.

Using the *his* leader peptide translation speedometer system we were able to distinguish seven Mac-Lac colony color phenotypes that we have named **a** (white), **b** through **f** (increasing dark shades of red) to **g** (dark red with bile salts precipitation visible under the colony) (Fig 4B). For example, when one of the seven consecutive His codons in the leader peptide was replaced by a stop codon, a **g** phenotype on Mac-Lac plates was observed. However, when translation is fastest through the *his* leader peptide (attenuation is occurring) we observed the **a** phenotype (Fig 4B).

In order to isolate mutants that result in faster translation through translation-slow codons in the *his* leader gene, the selectable marker *sacB* was employed. SacB is levan sucrase, which converts sucrose to levan polymers. In the periplasm of Gram-negative bacteria, levan accumulation results in cell death [40]. A selection strain was constructed in which the *hisD* gene was replaced by a *sacB* gene and a chloramphenicol resistance cassette (FCF) (Δ*hisD*::*sacB-*FCF) so that *sacB* expression is dependent on the *his* operon promoter and on failure of attenuation (Fig 4A). Slow translation of the *his* leader, which produces a **g** (Lac^+^) phenotype on Mac-Lac plates, results in cell death on 6% sucrose media (Suc^-^), whereas fast translation produces an A (Lac^-^) Mac-Lac phenotype and growth on 6% sucrose media (Suc^+^) (Fig 4A). The transition from an **a** through **c-f** to **g** white to pink to darkening red color phenotypes corresponds to increasing *lac* operon expression (Fig 4B). Intermediate **c**-**f** colony color translation phenotypes often have mucoid colonies on 6% glucose. A strain that is diploid for the region of the chromosome that includes the *his* operon was constructed that carries the *hisD::*Mud-*lac* indicator in one of the duplicated regions and Δ*hisD*::*sacB-*FCF construct in the other (Fig 4C)*.* This construct allows for the selection and screening of fast-translation mutants that have a Suc^+^ Lac^-^ phenotype; the diploidy eliminates survival of recessive mutants that alter the leader gene or attenuator.

### Isolation of *trans*-acting mutations that increase translation rates

A strain merodiploid for the *his* operon carrying the *hisD::*Mud-*lac* reporter in one *his* operon and the Δ*hisD*::*sacB*-FCF construct in the other *his* operon (above) with the slowly translated UCA-UAC codon pair replacing His4-His5 in both *his* leader genes was constructed. This strain has a Lac^+^ Suc^-^ growth phenotype due to failure of attenuation and high levels of transcription into both *his* operons. Selection for Suc^+^ and screening for Lac^-^ should isolate mutants that allow attenuation to occur in this strain (see Fig 3A). Four mutant types were expected to have a Suc^+^ Lac^-^ phenotype: (i) global regulatory mutants defective in transcription of the *his* operon promoter(ii) global regulatory mutants defective in translation of both *sacB* and the *lac* operon(iii) mutants that result in the inability to translate through the *his* leader peptide mRNA upstream of the seven consecutive His codons and result in attenuator formation, and (iv) mutants that result in an increase in translation speed through the UCA-UAC codon pair.

Selection for spontaneous Suc^+^ cells was performed with 15 independent cultures. A total of ~6 x 10^8^ cells from each culture was subject to Suc^+^ selection conditions yielding mutants at the relatively high frequency of ~1 in 10^5^ cells. This is a frequency typical for the loss-of-function of a single *Salmonella* gene. We presumed the majority of these did not affect attenuation and were defective in the Δ*hisD*::*sacB*-FCF locus such that SacB function was decreased or eliminated. Only 1/10^3^ of the Sac^+^ mutants had a Lac^-^ phenotype indicating that the mutation affected expression of both *his* operon constructs. These Suc^+^ Lac^-^ mutants were found to reside in four chromosomal linkage classes and DNA sequence analysis identified mutations in two genes encoding subunits of RNA polymerase (*rpoA* and *rpoD*) and two essential tRNA genes (*thrU* and *leuZ*).

### Global regulatory mutants in *rpoA* and *rpoD,* defective in transcription of the *his* operon promoter

Global regulatory mutants were expected that decrease transcription enough to lower *sacB* expression to a non-toxic level. Transcription is carried out by RNA polymerase, which has five subunits α_2_ββ’ω. RpoA is the α-subunit of RNA polymerase. The C-terminal domain of *Salmonella* RpoA (residues 249-329) utilizes the UP element of promoters to enhance recognition [41]. This region includes the Suc^+^ Lac^-^ *rpoA* (E261K) mutant suppressor that came out of the selection. We presume that the RpoA E261K substitution interferes with promoter recognition, thus resulting in decreased transcription of both *his* operons. The other transcription-affected mutant was isolated in *rpoD* (R605L). RpoD is sigma^70^, the primary promoter recognition factor in *Salmonella* Typhimurium. RpoD is commonly divided into four contiguous peptide regions, where Region 4 binds the -35 promoter region to allow recognition by RNA polymerase [42]. R506L in RpoD is in region 4 of the protein, which is expected to affect the ability of RpoD to recognize the -35 promoter regions of the two copies of the *his* operon promoter. Thus, both mutations likely decrease promoter recognition by RNA polymerase which leads to decreased transcription and translation, resulting in the observed Suc^+^ Lac^-^ phenotype.

### ThrU(C40A) results in a general defect in mRNA translation that is suppressed by gene amplification

**Fig 5:**
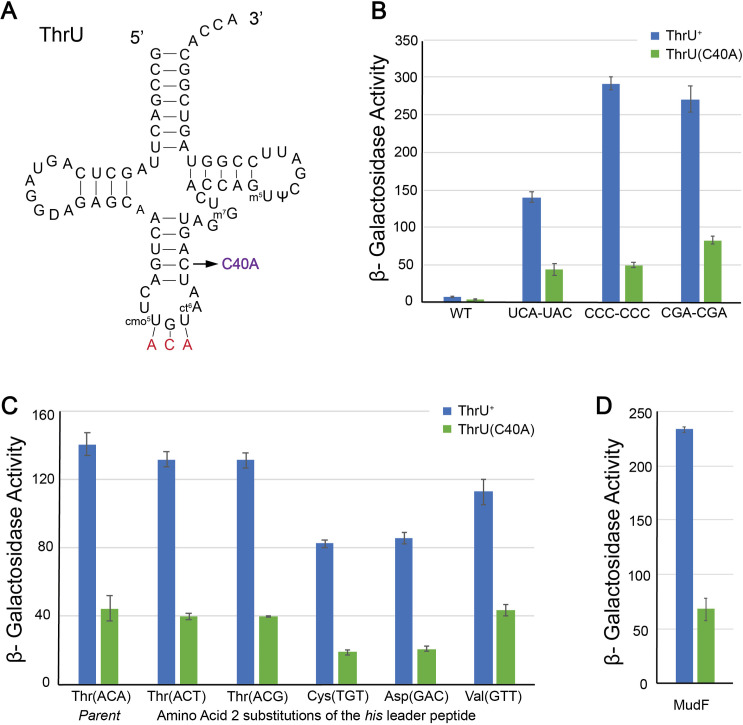
The ThrU(C40A) substitution results in *his* leader attenuation. **(A)** The RNA secondary structure of tRNA^ThrU^ showing known RNA base modifications. ThrU is an essential threonine tRNA, as it is the only tRNA to decode ACA codons (red). The C40A substitution in ThrU is indicated by an arrow. **(B)** Quantification of the effect of ThrU(C40A) on *hisD-lac* expression by β-galactosidase assay in strains with different codon pair substitutions at positions His4-His5 in the His leader peptide. The effects of the ThrU(C40A) allele on *his* leader translation speed compared to ThrU^+^ with different codon pairs at His4-His5 positions in the *his* leader peptide region was measured and quantified. Various *his* leader constructs were tested, WT *hisL* (TH2141, TH28797), *hisL*(His4-His5 = UCA-UAC*)* (TH27505, TH27798)*, hisL*(His4-His5 = CCC-CCC) (TH17685, TH28798) and *hisL*(His4-His5 = CGA-CGA*)* (TH28084, TH28799). One-tailed heteroscedastic t-test showed that all data sets were statistically different (P = <0.001) **(C)** Quantification of the effect of ThrU(C40A) on *hisD-lac* expression by β-galactosidase assay in strains with different codon substitutions at codon 2 in the His leader peptide. The effects of the ThrU(C40A) allele on *his* leader translation speed compared to ThrU^+^ with different codon pairs at the codon 2 position in the *his* leader peptide region was measured and quantified. Various *his* leader constructs were tested, WT *hisL*(codon 2 = ACA) (TH27505, TH27698), *hisL*(codon 2 = ACT) (TH29795, TH29800)*, hisL*(codon 2 = ACG) (TH29794, TH29799), *hisL*(codon 2 = TGT) (TH29792, TH29797), *hisL*(codon 2 = GAC) (TH29793, TH29798), and *hisL*(codon 2 = GTT) (TH29796, TH29801). One-tailed heteroscedastic t-test showed that all data sets were statistically different (P = <0.001) **(D)** The MudF transposon contains an intact *lac* operon with its native *lac* promoter-operator region. The effect of the ThrU(C40A) allele on *lac* operon expression independent of *his* operon expression was determined by measuring β-gal levels expressed from a MudF chromosomal insertion unlinked to the *his* operon (*zdx-3729::*MudF) (TH24401, TH28796). β-Galactosidase activities are listed in S3 Table. One-tailed heteroscedastic t-test showed that all data sets were statistically different (P = <0.001).

ThrU is the only tRNA that recognizes the ACA(Thr) codon, and it is an essential tRNA in *Salmonella*. The *thrU*(C40A) allele has a base substitution in the anticodon stem (Fig 5A). The ThrU(C40A) substitution results in the suppression of the slow translation phenotype with the UCA-UAC (Ser-Tyr) codon pair at His4-His5 in the *his* leader peptide. The effect of the ThrU(C40A) substitution is an approximately three-fold decrease in *hisD::*Mud-*lac* expression from 140 ± 13 β-Gal units in a *thrU*^+^ background to 44 ± 15 units in the *thrU*(C40A) background (Fig 5B). The ThrU(C40A) substitution represented a potential global regulatory mutant defective in translation of both *sacB* and the *lac* operon to produce the Suc^+^ Lac^-^ phenotype.

We tested if the ThrU(C40A) effect was specific to the *his* attenuation mechanism. If this were true, we expected that β-galactosidase expression in *hisD::*Mud-*lac* strains with different slow translating codon pairs in the *his* leader peptide should be reduced. We tested the effect of the ThrU(C40A) substitution on *hisD*::Mud-*lac* expression with leader peptides carrying either a CCC-CCC (Pro-Pro) or CGA-CGA (Arg-Arg) at His4-His5 in the *his* leader peptide*.* Proline is the only N-alkylamino acid (imino acid) among the 20 natural amino acids. It has been shown that translation of proline codons occurs at a slower rate because the cyclic proline residue constrains the secondary structure of the translating protein as compared to insertion of the other 19 amino acids [43]. Arginine codons are also translated slowly due to the positive charge interfering with transit through the exit channel on the ribosome [44]. The presence of the ThrU(C40A) substitution resulted in decreased β-Gal activities for both constructs. With the CCC-CCC codon pair at His4-His5, the levels of *hisD::*Mud-*lac* expression decreased from 290 ± 9 to 49 ± 3 β-Gal units. With the CGA-CGA codon pair at His4-His5, the levels of *hisD::*Mud-*lac* expression decreased from 270 ± 20 to 49 ± 3 β-Gal units (Fig 5B).

The sequence of the *his* leader peptide has a threonine codon, ACA, at amino acid 2. Previous work on the *his* leader peptide has shown that early termination of translation in the *his* leader peptide results in attenuation [37]. We hypothesized that either ThrU(C40A) decodes ACA poorly resulting in premature stalling and termination of His leader peptide translation preventing *hisD::sacB-*FCF and *hisD::*Mud-*lac* from being expressed or the ThrU(C40A) had an indirect effect on leader peptide translation independent of the ACA codon at amino acid 2. To test these possibilities, we replaced codon 2 of the His leader peptide with different codons that had a similar *hisD-lac* expression phenotype (Mac-Lac Red) as the original strain TH27505 (*hisL* amino acid 2 = ACA, His4-His5 = UCA(Ser)-UAC(Tyr)). Five codon replacements at amino acid 2 were tested: UCU (Cys), GAC (Asp), ACU (Thr), ACG (Thr) and GUU (Val). Unlike the UCA codon, which is only recognized by ThrU, the ACU (Thr) codon is recognized buy three tRNA species: ThrT, ThrU and ThrV, and the ACG (Thr) codon is recognized by ThrU and ThrX. The effects of the amino acid 2 substitutions on *hisD-lac* expression in the presence or absence of the ThrU(C40A) allele was determined by measuring β-gal levels and the results are shown in Fig 5C. For all five strains the presence of the ThrU(C40A) allele resulted in a similar decrease in β-gal levels (3- to 5-fold) as the parent strain. These results indicates that the effect of the ThrU(C40A) substitution is not due to a defect in translation at the ACA(Thr) codon at amino acid 2 of the His leader and likely results in a general defect in mRNA translation.

If ThrU(C40A) does decrease general translation of the *lac* operon in the *hisD::*Mud-*lac* fusion, we would expect reduced expression of a *lac* operon to not be under the control of the *his* operon attenuation control mechanism. *Salmonella* has no native *lac* operon, but we inserted a MudF element [45], which carries an intact *E. coli lac* operon, into a chromosomal location that is not linked to the *his* operon (*zdx-3729*::MudF). We tested the effect of the ThrU(C40A) substitution on *lacZ* expression from the MudF element. The β-Gal activity was reduced ~3-fold in the thrU(C40A) background (68 ± 11 β-Gal units) compared to β-Gal levels in the *thrU*^+^ strain background (240 ± 2 β-Gal units) (Fig 5B). This result demonstrates that the ThrU(C40A) effect was due to an overall reduction in cellular translation.

In working with the various *hisD-lac* fusion constructs in the *thrU*(C40A) mutant background, we observed a high reversion rate to a Lac^+^ and a larger-colony phenotype when streaked on Mac-Lac indicator plates. Strain TH27698 (*hisL10592*(His4-5 = TCA-TAC) *hisD993*::MudJ *thrU*(C40A)) was streaked onto two Mac-Lac plates and incubated at 30°C and 37°C. The frequency of Lac^+^, larger-colony phenotype mutants appeared to be greater at 37°C. Five independent cultures were started from small, Lac^-^ (Mac-Lac white) colonies and grown overnight at either 30°C or 37°C. The cultures were serially diluted, plated on Mac-Lac indicator plates and incubated at 30°C. Plates from cultures grown at 30°C yielded larger-colony, Lac^+^ mutants at frequencies of 10^-3^ to 10^-4^, while plates from cultures grown at 37°C yielded larger-colony, Lac^+^ mutants at frequencies of ~10^-2^. These mutant frequencies are those expected from chromosomal duplication events rather than chromosomal mutations. The genomes of four independent larger-colony, Lac^+^ mutants were sequenced and all four were found to be duplicated for the region of the chromosome covering the *thrU* locus (S1 Fig). It had been previously reported that this region of the chromosome experiences duplications at a frequency of ~10^-2^ when grown in LB medium at 37°C [46], which is consistent with our findings. This suggests that the ThrU(C40A) allele results in a temperature-sensitive, slow-growth phenotype due to reduced aminoacyl-ThrU in the cell, which can be compensated by increasing the gene dosage of the *thrU* locus in the cell.

### tRNA^LeuZ (A25G)^ does not affect translation of *lacZ* or *sacB*

**Fig 6:**
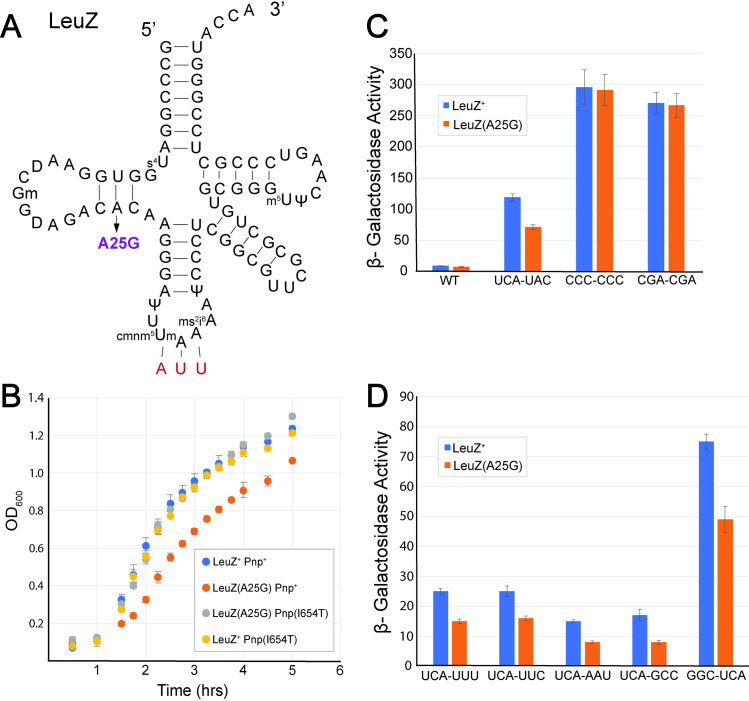
The tRNA^LeuZ(A25G)^ substitution results in faster translation speeds through the *his* leader peptide region containing UCA (serine) codons. **(A)** The secondary structure of tRNA^LeuZ^ showing known RNA base modifications. tRNA^LeuZ^ is an essential leucine tRNA. tRNA^LeuZ^ is the only tRNA that decodes UUA codons (Red). The *leuZ*(A25G) substitution is depicted by an arrow. **(B)** The effect of a *pnp*(I654T) allele on the growth rate of a strain with the tRNA^LeuZ(A25G)^ allele at 37°C. Growth rates were performed by diluting overnight cultures in fresh medium and measuring OD_600_ at 37°C until the OD_600_ was greater than 1. Wild-type *Salmonella* Typhimurium (Blue, TH437), *leuZ*(A25G) (Orange, TH27694), *leuZ*(A25G) *pnp*(I654T) (Gray, TH28496), and Pnp(I654T) (Yellow, TH28581) were all assayed for growth in LB medium at 37°C. The strain carrying the *leuZ*(A25G) allele exhibited a slower growth phenotype at 37°C in a *pnp*^+^ strain background. This slow-growth phenotype was suppressed by the *pnp*(I654T) allele. **(C)** β-Galactosidase assays were used to quantify the effect of tRNA^LeuZ(A25G)^ on hisD::Mud-*lac* expression. The *leuZ*(A25G) mutation suppressed the slow-translation phenotype of the *his* leader with His4-His5 replaced by UCA(Ser)-UAC(Tyr) (TH27505, TH27689), but did not suppress the slow-translation phenotype with His4-His5 replaced with either the CCC(Pro)-CCC(Pro) (TH17685, TH28497) of CGA(Arg)-CGA(Arg) (TH28084, TH28498) codon pairs. One-tailed heteroscedastic t-test showed that the WT and UCA(Ser)-UAC(Tyr) in *leuZ*^+^ and *leuZ*(A25G) backgrounds were statistically different (P = 0.01 and 0.001, respectively). The CCC(Pro)-CCC(Pro) and CGA(Arg)-CGA(Arg) results were not significantly different **(D)** The *leuZ*(A25G) mutation suppressed the stalled translation phenotypes of codon pairs containing the UCA codon in the *his* leader peptide system. In order to determine if the effect of tRNA^LeuZ(A25G)^ on the suppression of slow-translation was specific to translation through UCA codons, β-galactosidase levels were determined in strains containing the hisD:*:*Mud-*lac* reporter and several His4-His5 leader peptide constructs where the UCA codon was present at either His4 or His5. The *his* leader alleles that were assayed were *hisL10634*(His4-His5 = UCA-UUU; TH28865 (leuZ^+^) and TH28503 (leuZ(A25G)), *hisL10633*(His4-His5 = UCA-UUC; TH28866 (leuZ^+^) and TH28502 (leuZ(A25G)), *hisL10632*(His4-His5 = UCA-AAU; TH28867 (leuZ^+^) and TH28501 (leuZ(A25G)), *hisL10635*(His4-His5 = UCA-GCC; TH28868 (leuZ^+^) and TH28504 (leuZ(A25G)), and *hisL10636*(His4-His5 = GGC-UCA; TH28869 (leuZ^+^) and TH28505 (leuZ(A25G)). Cells were grown at 37°C. β-Galactosidase activities are listed in S3 Table. One-tailed heteroscedastic t-test showed that all data sets were statistically different (P = <0.001).

As mentioned above, the Suc^+^ Lac^-^ mutants included a *leuZ* mutant allele LeuZ(A25G). Genome sequencing revealed the presence of a second mutation in the structural gene of polynucleotide phosphorylase, *pnp*, resulting in an I645T substitution. When the *leuZ*(A25G) allele was separated from the *pnp* (I654T) allele, the cells grew poorly at 37°C. Thus, all further work was carried out in a *leuZ*(A25G) *pnp*(I654T) double mutant background. The *leuZ*(A25G) *pnp*(I654T) double mutant also resulted in a faster translation phenotype with the UCA-UAC codon pair at His4-His5 in the *his* leader peptide at 37°C (all substitutions discussed below are at this position). tRNA^LeuZ^ is an essential leucine tRNA that recognizes UUA and UUG codons. Polynucleotide phosphorylase (Pnp) is a 3’ to 5’ exoribonuclease. Pnp has been shown to degrade unstable uncharged tRNAs. Loss of Pnp increases the intracellular levels of precursor tRNAs [47–49]. The *leuZ*(A25G) allele was separated from the *pnp* allele and the resulting *leuZ*(A25G) *pnp*^+^ strain showed a poor-growth phenotype at 37°C (Fig 6B). We conclude that the *pnp*(I654T) substitution likely stabilizes the intracellular concentration of tRNA^LeuZ(A25G)^, thus allowing a normal growth phenotype at 37°C. Expression of the *hisD::*Mud-*lac* reporter with the UCA-UAC codon pair decreased from 130 β-gal units in a *leuZ*^+^ control to 85 in the presence of the *leuZ*(A25G) *pnp*(I654T) alleles (Fig 6C). We then tested the effect of the *leuZ*(A25G) *pnp*(I654T) mutations on *hisD*::Mud-*lac* expression with leader peptides carrying either a CCC-CCC (Pro-Pro) or CGA-CGA (Arg-Arg)*.* Contrary to the results obtained with tRNA^ThrU(C40A)^ (Fig 5), the presence of the *leuZ*(A25G) *pnp*(I654T) mutations had no effect on *hisD*::Mud-*lac* expression in both constructs. With CCC-CCC the levels of *hisD::*Mud-*lac* expression was 250 β-gal units in both the *leuZ*^+^ *pnp*^+^ and *leuZ*(A25G) *pnp*(I654T) strains. With CGA-CGA the levels of *hisD*::Mud-*lac* expression was 180 β-gal units in both *leuZ*^+^ *pnp*^+^ and *leuZ*(A25G) *pnp*(I654T) backgrounds (Fig 6B). These results suggest that the effect of *leuZ*(A25G) *pnp*(I654T) on translation through UCA-UAC codon pair was not due to a general defect in mRNA translation in the cell or an overall effect on the *his* attenuation mechanism as observed with the *thrU*(C40A) allele.

The possibility that tRNA^LeuZ(A25G)^ was defective in *sacB* translation was also tested to confirm that the Lac^-^ Suc^+^ phenotype of the *leuZ*(A25G) allele in our assay was not due to an overall defect in translation. There are 10 LeuZ-dependent UUA Leu codons within the coding region of *sacB* at positions 13, 90, 109, 177, 259, 277, 308, 384, 398 and 469. A defect in translating UUA codons by tRNA^LeuZ(A25G)^ could result in the Suc^+^ selection phenotype. We tested this hypothesis by replacing the 10 UUA codons in *sacB* with different leucine codons (CTN*)* that are not decoded by tRNA^LeuZ^*.* Sucrose phenotypes were assayed on 6% sucrose plates at 37°C in either a *leuZ*^*+*^ or a *leuZ*(A25G) *pnp*(I654T) background with this modified *sacB*(CTN) gene*.* The effect of *leuZ*^+^ and *leuZ*(A25G) alleles on *sacB* expression (low expression = Suc^+^ versus high expression = Suc^-^) was determined in the strain background with the UCA-UAC (Ser-Tyr) codon pair at His4-His5 in the *his* leader peptide and the *hisD* coding sequence replaced with *sacB* containing the Leu codon substitutions (Δ*hisD*::*sacB*(CTN)-FCF). The *leuZ*^+^ background strain remained Suc^-^; the *leuZ*(A25G) *pnp*(I654T) mutant background remained Suc^+^. Had the *leuZ*(A25G) *pnp*(I654T) mutant background become Suc^-^ we would have concluded that the Suc^+^ phenotype in our original selection was due to a defect in translating *sacB* and not faster translation through UCA-UAC in the leader sequence. We conclude that the *leuZ*(A25G) mutation does not result in a general decrease in translation of UUA codons in either the Δ*hisD*::*sacB*-FCF or Δ*hisD*::*sacB*(CTN)-FCF strain and that the presence of tRNA^LeuZ(A25G)^ results in faster translation through the UCA-UAC codon pair in the *his* leader sequence.

### The effect of tRNA^LeuZ(A25G)^ is specific to UCA codons

The above results suggest that the *leuZ*(A25G) allele does not have a direct effect on *his* leader attenuation, and that it results in an increased translation speed through the UCA-UAC codon pair in our speedometer assay. Previously, we reported the construction of 128 codon pair combinations at His4-His5 with UCA(Ser) held constant at either His4 or His5 in the *his* leader peptide sequence [39]. We tested whether the *leuZ*(A25G) allele could affect the apparent translation speed through other slow-translated codon pairs with UCA in the His4 or His5 position. In our previous measurements, apparent translation rates through UCA-UUU(Phe), UCA-UUC(Phe), UCA-AAU(Asn), UCA-GCC(Gly), GGC(Ala)-UCA replacements resulted in stalled translation when compared to a strain with the WT *his* leader sequence [39]. Rates were quantified using β-gal assays for these codon pair combinations in the presence of *leuZ*(A25G) *pnp*(I654T). For each codon pair that included UCA*,* stalled translation was rescued in the presence of tRNA^LeuZ(A25G)^ (Fig 6D), while all pairs without a UCA were not affected by tRNA^LeuZ(A25G)^ (Fig 6C). We conclude that the tRNA^LeuZ(A25G)^ rate enhancement effect on slow-translating UCA-UAC is specific to the UCA codon.

### tRNA^LeuZ(A25G)^ mediates leucine incorporation at UCA codons

**Fig 7:**
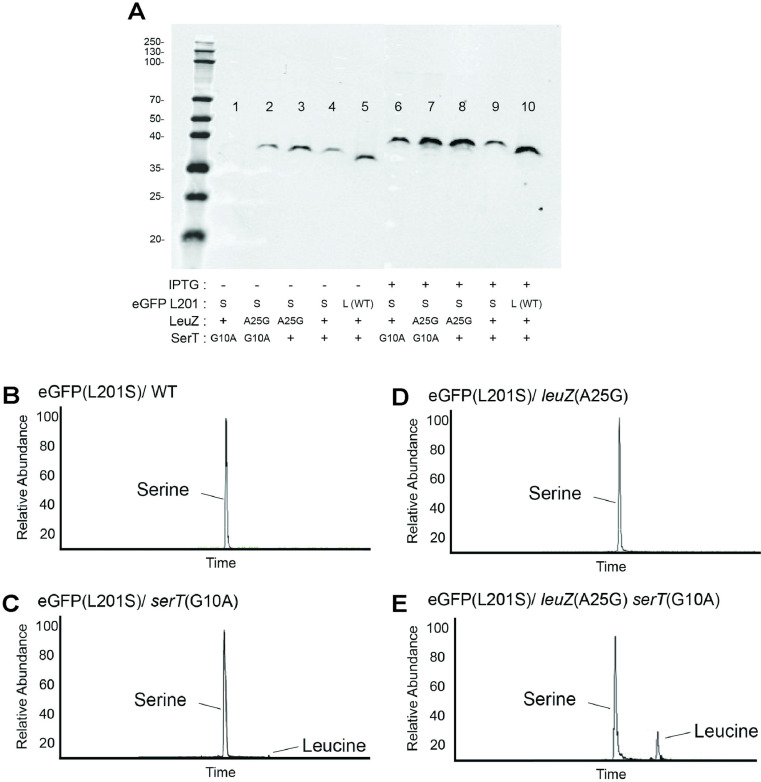
The effect of tRNA^LeuZ(A25G)^ on the incorporation of leucine at UCA codon 201 in eGFP(L201S). eGFP(L201S) was expressed and purified from leuZ+ serT(G10A), leuZ(A25G) serT(G10A), leuZ(A25G) serT+, and leuZ+ serT+ strain backgrounds. **(A)** eGFP expression patterns (western blots using anti-GFP antibody) without and with IPTG-induction conditions in different strain backgrounds indicated. **(B–E)** Isolated eGFP protein was digested with trypsin and peptides were analyzed by LC-MS/MS. **(B–C)** Only serine was present at amino acid position 201 of eGFP(L201S) in the wild-type (*leuZ*^+^ (**B**) and *leuZ*(A25G) (C)) mutant backgrounds. **(D)** There were two peptides isolated from the eGFP(L201S)/ *serT*(G10A) strain (TH28590). The major peak had serine incorporated at AA201, while the minor peak had leucine incorporated at AA201. The leucine peak is 1% relative to the serine peak. The two peptides containing Serine or Leucine were eluted at time 73.66 and 80.79 respectively. **(E)** There were two peptides isolated from eGFP(L201S)/ *serT*(G10A) *leuZ*(A25G) (TH28592). The major peak had serine incorporated at AA201, while the minor peak had leucine incorporated at AA201. The leucine peak is 20% relative to the serine peak. The two peptides containing Serine or Leucine were eluted at time 66.03 and 71.11 respectively.

If tRNA^LeuZ(A25G)^ is decoding UCA codons, it should cause misincorporation of leucine at serine codons. To test this hypothesis, we took advantage of the finding that a change in amino acid 201 of eGFP to serine (UCU) resulted in a nonfluorescent, unstable phenotype in yeast [50]. We found that a UCA serine substitution at amino acid 201 of eGFP had the same phenotype in *Salmonella* as the UCU substitution reported in yeast. The eGFP- and eGFP(L201S(UCA))-expressing genes were cloned into an IPTG inducible pTrc99a-10xHis-FLAG (Ap^R^) vector plasmid and introduced into *Salmonella* strains to characterize the effect of tRNA^LeuZ(A25G)^ on translation of the eGFP(L201S) gene. Under inducing conditions, the strains expressing eGFP were fluorescent while those expressing eGFP(L201S), were not fluorescent. The eGFP(L201S) substitution resulted in reduced expression levels, which is consistent with reduced stability (Fig 7A). The eGFP(L201S) also exhibited a slightly slower gel migration rate than the wild-type protein (Fig 7A). The eGFP(L201S) protein was purified from *leuZ*^+^ and *leuZ*(A25G) *Salmonella* strains, and mass spectrometric sequencing of the peptide region that includes AA201, showed that there was only serine present (Fig 7B and 7C). We reasoned that the inability to detect leucine incorporation in eGFP(L201S) was because the misincorporation event might be rare due to failure of the mutant tRNA to compete successfully with the native tRNA^SerT^ that normally translates the UCA codon. To increase the chances of leucine incorporation, the experiment was repeated in a tRNA^SerT^ mutant strain that is defective in translating the UCA codon. tRNA^SerT^ is the only tRNA that decodes the UCA serine codon. A tRNA^SerT(G10A)^ substitution was previously isolated in *E. coli* as a temperature-sensitive lethal mutant and was found to be important in cell cycle regulation [51]. In *Salmonella* Typhimurium, the *serT*(G10A) allele was isolated as a temperature-sensitive lethal mutant defective in translation of the UCA codon at permissive temperatures in the flagellar regulatory gene, *flgM* [52]. The presence of the *serT*(G10A) allele should increase the frequency of misincorporation of leucine into UCA codons by tRNA^LeuZ(A25G)^. The presence of the *serT*(G10A) allele resulted in a significant reduction of eGFP(L201S) levels in both the *leuZ*^+^ and *leuZ*(A25G) backgrounds (Fig 7A). His-FLAG-tagged eGFP(L201S) was purified from *leuZ*^+^ and *leuZ*(A25G) *Salmonella* strains containing the *serT*(G10A) allele. Analysis of their relevant peptides revealed that misincorporation of leucine at the UCA 201 codon in eGFP(L201S) increased significantly to 20% in the *serT*(G10A) *leuZ*(A25G) background relative to the *leuZ*^+^ control (the latter remained at <1% leucine) (Fig 7D and E). This result clearly shows that tRNA^LeuZ(A25G)^ can incorporate leucine at a UCA serine codon.

### Stalled translation results in eGFP (L201S) fluorescence in the presence of tRNA^LeuZ(A25G)^

Even though the mass spectrometry data demonstrated that tRNA^LeuZ(A25G)^ can incorporate leucine at UCA codon 201 of eGFP (L201S) in the above construct, not enough was produced to allow us to detect fluorescence. Given the strong effect of the tRNA^LeuZ(A25G)^ substitution on translation of the UCA codon in the *his* leader peptide system, we were surprised at the difficulty in detecting misincorporation of leucine in place of serine for eGFP(L201S) and the fact that the *serT*(G10A) allele is required to facilitate misincorporation. We have noted that the *his* leader peptide system is extremely sensitive to translation defects. For example, successive proline codons at His4-His5 in the *his* leader peptide gives the same loss of attenuation as a stop codon at either one of those positions ^34^. In other systems proline pauses are only detected with four consecutive prolines with an additional polyproline-specific elongation factor, EF-P, removed [39,53].

The *his* leader peptide system is unusual in that we also detect effects of codon changes on translation rates when the ribosome is after RNA polymerase during transcription-translation coupling. In the eGFP(L201S) suppression assay described above the majority of ribosomes translating the UCA codon at position 201 would not be the first ribosome that is coupled to RNA polymerase. Recent work from the Bustamante lab supports the assertion that the RNA polymerase coupled ribosome is unique [54]. In an in vitro transcription-translation coupled system, they found that the coupled, initial ribosome exerts mechanical force on the RNAP, effectively pushing it from behind, which resulted in an increased transcription speed at the expense of fidelity. The initial ribosome is unique in that it is physically connected to RNA polymerase through NusG protein [55]. Ribosome stalling in the *his* leader peptide’s consecutive His codons should result in a physical separation between the first ribosome and RNA polymerase and allow RNA polymerase to continue on into the attenuator stem-loop region. We hypothesized that failure to detect fluorescence of eGFP(L201S) with tRNA^LeuZ(A25G)^ and a much higher frequency of misincorporation of leucine at codon 201 of eGFP(L201S) was because we were not accounting for separation of RNA polymerase from the first ribosome as in the *his* leader peptide. It is possible that stalling of the first ribosome at UCA codon 201 in eGFP(L201S) might be mimicked by the presence of translation-elongation inhibitors, which might allow tRNA^LeuZ(A25G)^ time to be recognized by translating ribosomes not associated with RNA polymerase. Thus, slowing of translating ribosomes would phenocopy the effect of directly following RNA polymerase and provide time to recognize UCA by tRNA^LeuZ(A25G)^ bound to EF-Tu.

**Fig 8:**
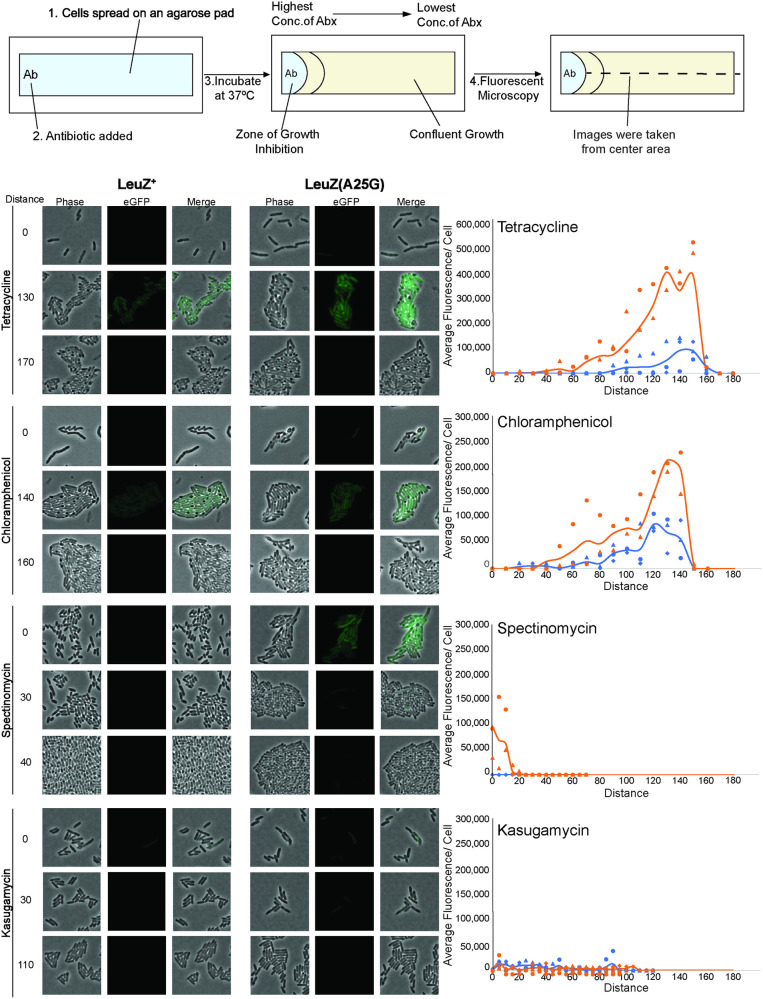
Stalling of translation-elongation resulted in increased fluorescence for strains expressing eGFP(L201S) in the *leuZ*(A25G) background. Incorporation of leucine at codon 201 in eGFP(L201S), resulted in a non-fluorescent phenotype. On a microscope slide, eGFP(L201S)/ *leuZ*(A25G) *pnp*(I654T) (TH29056) and eGFP(L201S)/ *leuZ*^+^ *pnp*(I654T) (TH29056 or TH29134) were plated onto an agarose pad. Antibiotics that inhibit either translation elongation (tetracycline, chloramphenicol, and spectinomycin) or translation initiation (kasugamycin) were spotted onto the far edge of the agarose slide. Images were taken every 5-10 steps starting at the edge of zone of inhibition, where each step is 0.89μm. Each assay was done in triplicate. Average eGFP fluorescence was quantified per cell for each replicate. The average between each replicate was calculated and plotted on the graph (solid line). The effect of each antibiotic on fluorescence was determined for both tRNA^LeuZ^ (Blue; ●,▲,◆) and tRNA^LeuZ(A25G)^ (Orange; ●,▲,◆) expressing strains.

There are numerous antibiotics that target the ribosome at different steps during translation [56]. We tested one translation-initiation inhibitor (kasugamycin) and three translation-elongation inhibitors (tetracycline, chloramphenicol and spectinomycin) on the ability of tRNA^LeuZ(A25G)^ to misincorporate leucine into codon UCA 201 in the eGFP(L201S) to produce detectable fluorescence. Intermediate concentrations of elongation inhibitors should slow ribosome progression through the cell. Exponentially growing cells were applied to a microscope slide with an agarose pad and antibiotic was applied to one end of the agarose covered slide. This allowed the antibiotic to form a concentration gradient by diffusion through the agar pad. A zone of cell growth inhibition was observed close to where the antibiotic was applied, but growth was observed at the lower subinhibitory antibiotic levels and throughout the rest of the slide. With each elongation inhibitor, but not with the initiation inhibitor, we observed a zone containing fluorescent cells (Fig 8). We suspect that the concentration of antibiotic in this zone was sufficient to cause an effect on translation of codons that was similar to the effect of separation of the leading ribosome from RNA polymerase resulting in high enough levels of mis-incorporation of leucine at UCA codon 201 of eGFP(L201S) by tRNA^LeuZ(A25G)^.

The results presented in Fig 8 showed a significant increase in fluorescent eGFP(L201S)-expressing cells in the *leuZ*(A25G) background as compared to *leuZ*^+^ for translation-elongation inhibitors tetracycline, chloramphenicol and spectinomycin. The effect of spectinomycin was seen close to the edge of the zone of inhibition while the effects of tetracycline and chloramphenicol were further from the edge. This may be due to differences in sensitivity of *Salmonella* to the different antibiotics. The translation-initiation inhibitor kasugamycin had no effect on leucine mis-incorporation (Fig 8).

## Discussion

We showed that an A25G substitution in the D-stem of the essential leucine tRNA^LeuZ^ is able to suppress the slow translation speed through tandem UCA-UAC codons at His4-His5 in the *his* operon leader sequence. The *leuZ*(A25G) allele is also able to increase the apparent translation speed through different codon pairs at His4-His5 in the *his* leader peptide system as long as one of the codons is UCA, but it does not affect the apparent translation speed through codon pairs that did not include a UCA codon. The *his* leader peptide does not contain leucine codons leading us to hypothesize that tRNA^LeuZ(A25G)^ is able to recognize UCA in the ribosome decoding center. Consistent with this hypothesis, we showed that leucine can be incorporated into eGFP with UCA at codon 201 in a strain expressing tRNA^LeuZ(A25G)^. This result suggests that the mutant tRNA^LeuZ(A25G)^ has an increased decoding capacity beyond the UUA and UUG codons recognized by wild-type tRNA^LeuZ^.

**Fig 9:**
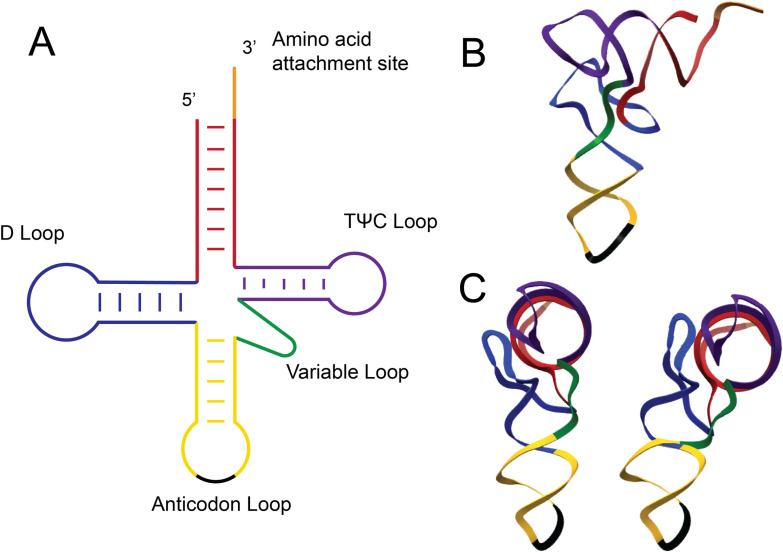
Model depicting straight versus bent tRNA structures. **(A)** A classical color-coded “cloverleaf” tRNA 2° structure is shown depicting the acceptor stem (red), the D-stem and loop (blue), the anticodon stem and loop (yellow), the variable loop (green) and the TΨC-stem and loop (purple). **(B)** A three-dimensional color-coded tRNA inverted L-shaped tRNA structure. **C.** Straight and bent three-dimensional tRNA structures presented at a 90° angle from the structure shown in **B**.

How might the A25G substitution in the D-stem of tRNA^LeuZ^ lead to extended decoding capacity and increased translation speed through UCA codons? tRNA species are often presented in two dimensions as “cloverleaf” 2° structures depicting the acceptor stem, the D-stem, and loop, the anticodon stem and loop, and the TΨC-stem and loop (Fig 9A). A tRNA’s three-dimensional structure forms an inverted L-shape (Fig 9B). The D-loop and TΨC-loop interact at the elbow region of the tRNA 3° structure. During tRNA recognition and prior to its acceptance as the correct tRNA for a specific codon, the ribosome undergoes a conformational change that leads to a bending of the tRNA at the elbow (Fig 9C). The bending of the cognate tRNA is coupled to hydrolysis of EF-Tu bound GTP, which is followed by accommodation of the correct aminoacyl-tRNA or rejection of an incorrect one [18,27,28]. This GTP hydrolysis step in A-site recognition provides a final fidelity check to ensure that the correct tRNA is chosen for that specific codon. If a non-cognate tRNA is present in the A-site during the GTP hydrolysis step, the tRNA is rejected due to the mismatch in the codon-anticodon interaction [10,18,21]. Near cognate tRNAs that differ by only one base mismatch in the anticodon-codon interaction can remain in the A-site longer than a non-cognate interaction until the GTP hydrolysis step in codon recognition. In order to discriminate near-cognate from cognate tRNAs, the ribosome relies on the final recognition step that includes the bending of the tRNA in the D+TΨC-stem interacting (hinge) region near the elbow of the tRNA structure [28,57] (Fig 9).

**Fig 10:**
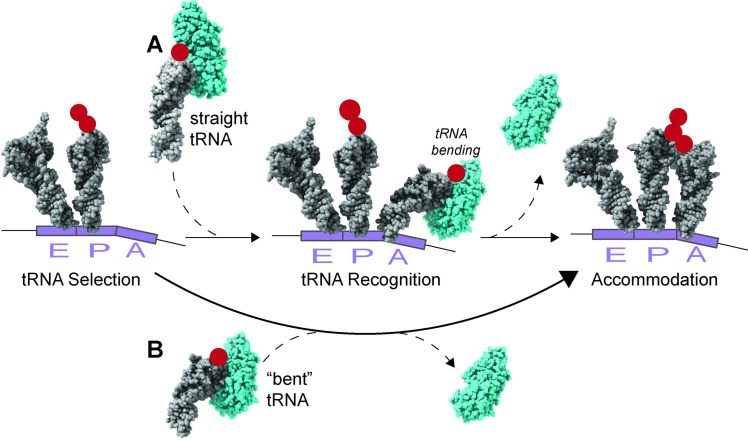
Model for near-cognate codon recognition by pre-bent/flexible tRNAs. **(A)** During translation, EF-Tu bound to GTP and aminoacylated-tRNA enters the A- site of the decoding center straight. As the codon- anticodon interaction is being assessed, the ribosome changes conformation resulting in a bent tRNA structure. After recognition of cognate tRNA, GTP is hydrolyzed to GDP and EF-Tu dissociates from the ribosome, which allows for accommodation of the tRNA into the A-site fully. **(B)** A mutation in the hinge region of a tRNA that results in a bent tRNA structure bypasses the final fidelity check in codon recognition. When the mutant, bent tRNA enters the ribosome A-site, GTP is hydrolyzed faster than the ribosome allowing the tRNA to insert an incorrect amino acid with a near-cognate codon. Thus, evolution has provided a mechanism to increase fidelity for codon recognition, but at a cost of translation speed. Structures adapted from [13,14]. Structures were taken from the Protein Data Bank. More specifically, PDB files: 4V68, 4V6F, 4V5S, and 4V5R. tRNA selection and tRNA recognition from [14], tRNA accommodation from structures in [70], and bent tRNA and straight tRNA came from structures in [15].

One possible way to suppress the need for a complementary anticodon/ codon interaction is to have a pre-bent or more flexible tRNA [13]. If a near-cognate tRNA that is more flexible enters the A-site, the ability of the ribosome to hydrolyze GTP and accommodate the tRNA is faster than its ability to sense an incorrect near-cognate tRNA in the A-site [58] (Fig 10). There exists a single example of this in the literature: the “Hirsh suppressor” in *E. coli* tRNA^TrpT^. In 1971 Hirsh isolated a G24A D-stem substitution in tRNA^TrpT^ as a UGA nonsense suppressor that allowed recognition of both the UGG(Trp) and the UGA nonsense codons [15,59]. It was later shown that translation with tRNA^TrpT(G24A)^ exhibits an accelerated forward reaction rate and structural data revealed that tRNA^TrpT(G24A)^ has a stable bent conformation [15,58]. The genetic data presented here predicts that tRNA^LeuZ(A25G)^ results in a structural change in the tRNA hinge region similar to that in tRNA^TrpT(G24A)^ that allows recognition of the near-cognate UCA(Ser) codon resulting in an increase in translation speed in the *his* translation speedometer assay. Our findings suggest that this may be a general phenomenon and that base substitution mutants in the hinge region of other tRNA species might also increase translation speed through near-cognate codons and result in amino acid misincorporation.

Translation is fine tuned to balance speed and accuracy. Amino acid misincorporation during translation in *E. coli* has been shown to occur at a frequency of ~10^-3^-10^-4^ [60,61]. Hyper-accurate ribosomal mutants, initially isolated in the 1960s, were found to result in a reduction of growth rates due to a reduction in translation speed that was concomitant with increased translational accuracy [62,63]. The translation machinery has evolved to balance the relative costs of translation speed and fidelity. The study presented here demonstrates that it is possible to select tRNA mutants that change this balance and allow for faster translation speed at the cost of fidelity.

One striking result obtained in this study was that only the *thrU*(C40A) mutant was obtained that was defective in overall mRNA translation out of ~10^10^ cells were obtained. The UCA-UAC codon pair used for selection has a translation-slow phenotype similar to a stop codon in our *his* leader peptide system (a “**g**” Mac-Lac and sucrose-sensitive phenotype in Fig 4B) [39]. It is possible that selection for faster translation using codon pairs with less stringent selection phenotypes would allow for the isolation of overall translation-defective mutants. Otherwise, the *leuZ*(A25G) allele would likely not have been obtained by our genetic selection method. It is noteworthy that the *leuZ*(A25G) was isolated together with the *pnp*(I654T) allele. Transduction of the *leuZ*(A25G) into a *pnp*^+^ background resulted in poor growth at 37°C and no growth at 42°C. It is possible that inclusion of a *pnp*-defective allele into the original selection strain would have allowed for the isolation of other general mRNA translation tRNA mutants besides the *thrU*(C40A) allele. Another important result was the requirement of the *serT*(G10A) allele with the *leuZ*(A25G) to detect leucine incorporation at UCA codon 201 of eGFP. We hypothesize that this represents the difference in mRNA translation in the *his* leader peptide system as compared to translation of eGFP mRNA. Translation in the *his* leader peptide system only occurs where the translating ribosome is coupled to transcription by RNA polymerase. For eGFP gene translation, only the initial translating ribosome is coupled to transcription by RNA polymerase. All but the initial translating ribosomes are not directly behind RNA polymerase during translation of eGFP mRNA. A recent study reported that in bacteria where transcription and translation are coupled, the initial ribosome trailing RNA polymerase speeds up RNA polymerase at the expense of transcript fidelity via force and allostery [54]. If the reciprocal is true (RNA polymerase impedes trailing ribosomes), the speed of the translating ribosome in the *his* speedometer assay may be slowed by RNA polymerase and may explain the apparent amplification effect of tRNA^LeuZ(A25G)^ on the speed through UCA codons and the misincorporation of leucine relative to translation effects on ribosomes that follow the initial translating ribosome.

Provided that the ribosome can move faster through codons than RNA polymerase can transcribe DNA, it would be advantageous to have a mechanism to simultaneously enhance fidelity and reduce translation speed that would prevent RNA polymerase from incorporating incorrect bases and reduce the frequency of ribosome collisions during translation. We propose that the final fidelity check coupled to GTP hydrolysis at EF-Tu provides such a mechanism.

In addition, data presented in Fig 8 data showed that even in the *leuZ*^+^ background significant fluorescence was observed in sublethal concentrations of tetracycline and chloramphenicol. This result raises the possibility of mechanisms where, under conditions of stress, a cell could express a translation-elongation inhibitor to a level that would allow a high degree of misincorporation resulting in transient production of a plethora of altered proteins without mutation at the DNA level. Such a mechanism might provide an evolutionary survival advantage to the organism.

## Materials and Methods

### Bacterial strains, plasmids, and media

All strains used in this study not designated as *E. coli* were derived from *Salmonella enterica* serovar Typhimurium wild-type strain LT2 and are listed in S1 Table. The list of DNA oligonucleotides used for strain constructions are listed in S2 Table. Cells were cultured in lysis broth (LB: Bacto-tryptone 10g/l, Bacto-yeast extract 5g/l, NaCl 5 g/l). Antibiotics were added to LB at the following final concentrations: 100 µg/ml sodium ampicillin (Ap), 12.5 µg/ml chloramphenicol (Cm), 15 µg/ml tetracycline-HCl (Tc), 50µg/ml kanamycin sulfate (Km), 100 µg/ml carbenicillin. Apex agar (12g/l) was used for preparation of solid medium. Selection of tetracycline-sensitive (Tc^S^) recombinants was done on zinc-fusaric acid selection media [64]. LB-arabinose was supplemented to 0.2% (w/v) as needed. Sucrose plates used the same recipe as above with a couple of minor changes, 60g/l of LB-sucrose and no NaCl added. Minimal E salts medium supplemented with 0.2% glucose was used as a minimal medium. supplements were added to minimal medium to a final concentration of the following compounds: 2 µg/ml X-Gal and 1.55 µg/ml histidine. Phenotypic lactose activity was observed using MacConkey lactose indicator medium (Mac-Lac), and 3,4,5-triphenyltetrazolium chloride-lactose (TTC-Lac) indicator medium supplemented with 0.2% arabinose if required.

### Transduction and λ-red recombination for strain construction

The generalized transducing phage of *S.* Typhimurium P22 *HT105/1 int-201* was using in all transductional crosses [65]. P22 phage lysates were prepared on donor strains. Briefly, 100ul of a P22 transducing lysate, diluted to 10^-6^ to 10^-7^ plaque forming units (PFUs) per ml, was mixed with 100ul of an overnight culture (2 x 10^8^ cells/ml) of the recipient strains. The mixture was allowed to incubate at either 37°C for 30 minutes or 30°C for 30 minutes for temperature sensitive mutants. The mixture was then plated onto selective media. The MudJ and MudA *lac* operon fusion elements were mobilized for strain construction by P22-mediated transduction selecting for kanamycin (Km^R^) or ampicillin resistance (Ap^R^) respectively. λ-red recombination was used for allele construction using insertion and replacement of the *tetRA* cassette from transposon Tn*10* following the protocol provided in [64]. Replacement of a *tetRA* cassette either by λ-red recombination or P22-mediated transduction was done by selecting for fusaric acid resistance as described [64]. To avoid background spontaneous tetracycline sensitive (Tc^S^) mutants in P22 transductional crosses to fusaric acid resistance, the P22 transducing lysates are diluted to ~10^8^ pfu/ml, let sit at either room temperature for one hour or incubated at 37°C for 30 minutes, and diluted 1000-fold prior to selection. This typically yields 100- to 1000-fold more Tc^S^ colonies than a no phage control cross. Tc^S^ colonies are then purified on green indicator plates [65] and genetic markers are verified by PCR or DNA sequence analysis. Gene replacements with the chloramphenicol acetyl transferase (*cat*) chloramphenicol resistance (Cm^R^) cassette from plasmid pKD3 [66] were also done following the protocol provided in [64]. Insertions of the FRT-Cm^R^-FRT (FCF) cassette from plasmid pKD3 [66] were also made following the protocol provided in [64] and used as linked selectable markers for strain construction purposes. The Δ*STM1941*::*tetRA* deletion/insertion and the Δ*STM1941*::*cat* deletion/insertion were constructed in a leuZ(A25G) background and used to move the leuZ(A25G) allele into various strain backgrounds by P22-mediated transduction selecting for tetracycline (Tc^R^) or chloramphenicol resistance (Cm^R^), respectively. The *argG*::Tn*10d*Cm allele was used as a linked marker to move *pnp* alleles various strain backgrounds by P22-mediated transduction selecting for Cm^R^. The *hpaB*::Tn*10d*Tc allele used as a linked marker to move *serT* alleles various strain backgrounds by P22-mediated transduction selecting for Tc^R^. The Δ*STM4140*::*tetRA* deletion/insertion was constructed in a thrU(C40A) background and used to move the leuZ(A25G) allele into various strain backgrounds by P22-mediated transduction selecting for Tc^R^. If the plasmid pSim-5 or pSim-6 was used for λ-red recombination, the only alterations to the protocol were as follows. Cells were grown in LB-Cm (pSim-5) or LB-Ap (pSim-6) at 30°C until OD600= 0.2. The culture was placed on ice for 5 minutes, and then heat shocked at 42°C for 15 minutes to induce expression of the λ-red genes.

### Randomization of the His2 and His3 codons upstream of UCA-UAC at His4-His5

A DNA fragment containing NNN His3 upstream of His4-His5 was generated using 4 cycles fill-in reaction between primers 8125 (his345-NNN-Ser-tyr) and 8000 (hisleader-rv), followed by 9 cycle amplification of the fill-in DNA fragment using primers 7999 (hisleader-fw) and 8000 (hisleader-rv). The DNA fragment containing NNN His2 upstream of His4-His5 was produced similarly using primers 8484 (His2NNNSerTyr)/8000 (hisleader-rv)/ 8484 (His2NNNSerTyr). Phusion DNA polymerase was used for all fill-in and DNA amplification reactions. The resulting DNA fragments were purified by ethanol precipitation and electroporated into electrocompetant strain TH15671 (pKD46/*hisO10508*::*tetRA hisD9953*::MudJ), previously grown with arabinose at 30°C. Following electroporation, the cells were resupended into 1mL LB media and incubated at room temperature for 1h, before being plated onto Tc^S^ plates at 37°C. Tc^S^ recombinants were patched onto Tc^S^ and MacConkey-Lactose plates (to assess expression of *hisD9953*::MudJ). The Lac^-^ phenotype recombinants were purified and pooled together. The Lac^-^ culture was diluted to approximate an overnight OD and genomic DNA was extracted using the Qiagen DNeasy Blood and Tissue Kit. A 20 cycle PCR reaction was performed using the genomic DNA template using primers 8256/8257 and Phusion polymerase. The purified PCR was submitted for amplicon sequencing (GeneWiz) to identify at once the identity of the Lac^-^ phenotype recombinants. Lac^+^ phenotype recombinants were purified and sequenced individually.

### Randomization of amino acid 2 (Thr) of the *his* leader peptide

Amino acid 2 of the *his* leader peptide was randomized using a similar strategy as described above. A *tetRA* element was first inserted at amino acid 2 of the *his* leader in strain TH27505 (*hisL10592*(His4-5 = TCA-TAC) *hisD9953*::MudJ) by λ-red recombination (with primers 10331/10858). A DNA fragment containing NNN instead of the amino acid 2 of the *his* leader was produced using a fill-in reaction between primers 9827 and 10859, followed by 9-cycles amplification of the fill-in reaction using primers 10864 and 8094. The DNA product was electroporated into the strain constructed above (containing the *tetRA* element at amino acid 2 and the λ-red plasmid pSim6). Tc^S^ recombinants were streaked directly onto Mac-Lac plates and the Lac^+^ colonies similar color phenotypes to the parent containing His4-5 = TCA-TAC (TH27505; red on Mac-Lac) were further purified and sent to DNA sequencing analysis.

### Mud-generated duplication of *his* operon

The duplication of the *his* operon was made using a strategy described in [67]. P22 phage lysates was grown on *hisD9953*::MudA (TH653) and on *fliN5103*::MudB (TH2094). Equal volumes of each phage lysates were incubated with an LT2 (TH437) overnight culture at 37°C for 30 minutes. Mud-containing transductants were selecting for on LB-Ap medium and Ap^R^ transductants were screened for prototrophic growth on minimal-glucose (E-dex) medium. Ap^R^ E-dex^+^ colonies were single colony purified and then streaked on minimal-Xgal medium and screened for blue-white segregation. Blue-white color segregation in the absence of Ap^R^ selection indicated that the region of the chromosome between the sites of insertion of the *hisD9953*::MudA and *fliN5103*::MudB elements had been duplicated yielding strain TH27503. When selection is not held for the duplication, the duplication is spontaneously lost resulting in a mixture of blue and white single colonies on minimal medium with X-gal [67]. The duplication from TH27503, was moved by P22-mediated transduction into Δ*his-3050* (TH85) selecting for Ap^R^, resulting in strain TH27504. The Δ*his-3050* allele is a non-transducible deletion of the *his* operon. Within the duplication of TH27504, the *his* leader construct was changed with His4-His5= UCA-UAC (TH27505) via P22-mediated transduction by selecting for Km^R^ and screening for blue-white color segregation E-dex-Xgal medium lacking Ap resulting in strain TH27506. The duplication in TH27506 was moved by P22-mediated transduction into strain TH27507 (*hisL10592* Δ*hisD10591*::*sacB*-FCF) selecting for Km^R^ and screening for blue-white color segregation E-dex-Xgal medium lacking Ap to produce strain TH27508.

### Selection for fast-translation mutants

Strain TH27508 has the His4-His5 codons of the *his* leader peptide region replaced with codons UCA-UAC in each duplicated copy. One duplicated region carries a *hisD9953*::MudJ allele, which results in Lac^+^ phenotype on Mac-Lac medium. The other duplicated region of the *his* operon contains the Δ*hisD10591*::*sacB*-FCF allele, which results in a Suc^-^ phenotype on 6% sucrose medium. Ten independent cultures of TH27508 were grown overnight in LB-Km at 37°C. A 0.1ml portion from each overnight culture was plated onto 6% sucrose-Km media and incubated overnight at 37°C. The following day each 6% sucrose-Km plate yielded ~1500 Suc^+^ colonies. Suc^+^ colonies were replica printed to Mac-Lac-Km medium and screened for those with a Lac^-^ phenotype. Suc^+^ Lac^-^ colonies were purified on Km-containing media and then either mapped or subject to next generation sequencing.

### Next generation sequencing

Genomic DNA from overnight cultures was isolated using the Qiagen DNA Blood and Tissue Kit. Next Generation Sequencing analysis was performed either using Nanopore Sequencing (Plasmidsaurus) or Illumina (150x150 bp Novaseq Sequencing) using Ilumina DNA prep for small genomes at the University of Utah Core Facility. Sequences were aligned to *Salmonella* Typhimurium LT2 genomic sequence using the program Geneious. SNP alleles were identified in strains TH27509 and TH27511 as *thrU*(C40A) and *rpoA*(E261K), respectively. Strains TH27509 and TH27511 were subject to NexGen sequencing and found to harbor the *thrU*(C40A) and rpoA(E261K) alleles, respectively. For the four independent larger-colony, Lac^+^ mutants from strain TH27698 (*hisL10592*(His4-5 = TCA-TAC) *hisD993*::MudJ *thrU*(C40A)), one of the four genomes (suppressor 1) was subject to Nanopore Sequencing (Plasmidsaurus) and the other three were sequenced using Illumina Sequencing (University of Utah Core Facility) for comparative purposes (S1 Fig).

### Isolation of Tn*10d*Tc insertions near the chromosomal *rpoD* and *leuZ* loci

Mutations in TH27510 and TH27512 were mapped using a linked Tn*10d*Tc transposon. P22 was grown on strain TT10423, which carries the Tn*10d*Tc insertions in the F’ plasmid and used to transduce strain TT10427 to Tn*10d*Tc-encoded Tc^R^ on LB-Tc medium. Strain TT10427 carries plasmid pNK972, which constitutively expresses the Tn*10* transposase gene. Because there is no F’ plasmid DNA in strain TT10427, it is not possible to inherit the Tn*10d*Tc transposons by homologous recombination. The Tc^R^ transductants are inherited by transposition into the recipient chromosome. More than 50,000 Tc^R^ transductants were pooled together, and P22 lysates were prepared on the pooled cells that carried insertions located at random positions through the chromosome. To isolate linked Tn*10d*Tc insertions to alleles later shown to be in the *rpoD* and *leuZ* genes, the pooled Tn*10d*Tc lysate was used to transduce strains TH27510 and TH27512, respectively, to Tc^R^ by plating on LB-Tc plates. Following overnight incubation at 37°C, the Tc^R^ colonies were screened for Lac^+^ on Mac-Lac medium. Co-transductional analysis of several linked insertions identified one Tn*10d*Tc allele in the *STM3216* locus that was 68% linked by P22-mediated transduction to the Lac phenotype associated with the *rpoD* mutant allele. Three Tn*10d*Tc insertions were found to be in the *STM1939*, *STM1941*, and *uvrC* genes and were 84%, 94% and 84% linked, respectively, to the Lac phenotype associated with the *leuZ* mutant allele. PCR amplification of the strain carrying the closely linked insertions with a primer that would recognize the ends to Tn*10d*Tc gave a product which after DNA sequence analysis revealed the Tn*10d*Tc insertion to be in the loci mentioned above.

### β-Galactosidase assays

Thirty µl of overnight cultures were each sub-cultured into 3 ml of fresh LB medium. Sub-cultures were incubated under aeration conditions at 37°C and grown to mid-log cell growth density (OD_600_ = 0.4). For strains that carry the *thrU*(C40A) allele, cells were incubated with aeration at 30°C and grown to mid-log (OD_600_ = 0.4). Following incubation on ice, cultures were pelleted by centrifugation, and cell pellets were resuspended in 3 ml of iced cold buffered saline. 0.5 ml culture samples (diluted if necessary) were added to 0.55 ml of Z-buffer (8.54 mM Na_2_HPO_4_, 5.5mM NaH_2_PO_4_•H_2_O, 0.75mM KCl, 0.25mM MgSO_4_•7H_2_O, pH 7.0) plus 5 ul of 10% sodium dodecyl sulfate and 100μl of chloroform. 0.2 ml of ONPG (4mg/ml) was added to each tube periodically. Reactions were stopped after 10 mins with the addition of 0.5 ml of 1M NaCO_3_. OD at wavelengths 420 and 550 were measured. Cell cultures were warmed to 30°C and their OD_650_ was measured. For each strain, the β-galactosidase activities reported are the average from at least three independent cell cultures started from different single colony isolates. β-galactosidase activity was expressed in nmol/min/OD_650_ and determined as described [68]. The one-tailed heteroscedastict-test was used for the statistical analysis shown in Figs 5 and 6.

### Identification of a secondary *pnp*(I654T) mutation in the *leuZ*(A25G) mutant strain

The *leuZ*(A25G) allele linked to a Δ*STM1941*::*tetRA* (Tc^R^) marker was introduced via P22-mediated transduction into strain TH27505 (*hisL10592*(His4-His5=TCA-TAC) *hisD9953*::MudJ) selecting Tc^R^. Tc^R^ transductants were sequenced for the *leuZ* region and one containing the *leuZ*(A25G) allele was kept as strain TH27691. Strain TH27691 had a noticeable growth defect at 37°C, indicating that a second mutation was present in the original background that suppressed the temperature-sensitive growth phenotype. A 2ml overnight culture of the parent strain TH28496 was prepared, genomic DNA was extracted using the kit and protocol from Qiagen DNeasy Blood and Tissue kit. Genomic DNA was then sent to the University of Utah’s core facilities for whole genome sequencing. The sequencing results were then aligned to the LT2 genome using the program Geneious. A SNP in the polynucleotide phosphorylase gene was identified as the *pnp*(I654T) allele, which was shown to be responsible for suppressing the temperature-sensitive growth defect caused by the LeuZ(A25G) substitution.

### The effect of *pnp*(I654T) on strain growth phenotypes

Growth curves were determined for the strains carrying the following mutant alleles: none (wildtype strain LT2), *leuZ*(A25G), *leuZ*(A25G) *pnp*(I654T), and *pnp*(I654T). Three independent cultures of strains LT2, TH27694 (Δ*STM1941*::*tetRA leuZ*(A25G)), TH28496 (*leuZ*(A25G) *pnp*(I654T)), and TH28581 *argG*::Tn*10d*Cm *pnp*(I654T)), were grown overnight at 30°C. The overnight cultures were sub-cultured into 3 ml of fresh LB medium and incubated under aerobic growth conditions at 37°C. The OD_600_ for each culture was measured every 30 min until cultures reached OD 0.4 and every 15 minutes after that until the OD_600_ measurement reached >1.0. Measurements obtained for each replicate were averaged and plotted using Excel.

### TTA to CTN codon replacement in SacB

The 10 UUA leucine codons (at positions 13, 90, 109, 177, 259, 277, 308, 384, 398 and 469) were replaced by other leucine codons (CTN) using λ-Red recombination in two steps. First, codons 13 through 177 of *sacB* were replaced by a *tetRA* element using primers 9496 and 9497 into a strain containing the *sacB* gene integrated into the chromosome of *Salmonella* (Δ*hisD10591*::*sacB*-FCF). Two PCR amplifications of *sac*B DNA with primer pairs 9479/9480 and 9481/9482 were produced. The resulting DNA fragments have a 24 bp overlap and were mixed together without primers followed by a 10-cycle PCR reaction, allowing the fragments to stitch together. A final 15-cycle PCR reaction on the stitched product, using external primers 9609 and 9610 was used for the λ-Red substrate product to replace the *sacB*::*tetRA* (ΔAA13-77) insertion sequence. The resulting strain had UUA leucine codons at positions 13, 90, 109 and 177 of *sacB* changed to CTC, CTG, CTG and CTG, respectively.

The second step consisted of inserting a *tetRA* element (made with primers 9483/9484) deleting amino acid codons 259 through 469 of *sacB* in the strain constructed above. First, Leu UUA codons at positions 259 and 277 of *sacB* were changed to CTG and CTC using primers 9485/9486 and *sacB* DNA as template. This PCR reaction was used as a template for another PCR reaction using the external primer pair 9487/9488. Second, changes of Leu UUA codons at positions 308 and 384 of SacB to CTA and CTC were generated by PCR using the primer pair 9489/9490 and *sacB* DNA as the template. Third, changes of Leu UUA codons at positions 398 and 469 of *sacB* to CTC and CTC were generated by PCR reaction using the primer pair 9491/9492 and *sacB* DNA as the template. The resulting three PCR products above were mixed in equimolar ratio, stitched together, and amplified (15 cycles reaction) with external primers 8487 and 9519, yielding a 711 bp DNA fragment encoding changes of Leu UUA codons at positions 259, 277, 308, 384, 398 and 469. This DNA fragment was used to replace the *tetRA* element deleting codons 259 through 469 of *sacB*.

### e GFP plasmid construction

Chromosomal insertion of the eGFP and eGFP(L201S) coding sequences were previously constructed at the *hisD* locus of S. Typhimurium by λ-red recombination. These strains were available in the lab’s strain collection. Primers 10149/10150 were used to PCR amplify the *eGFP* DNA region from strain TH28176 and the *eGFP*(L201S) DNA region from strain TH28397. Both PCR DNA products were purified using the Qiagen QIAquick PCR purification kit following the provided protocol. Strain TH27367 contains a ptrc99A-based plasmid derived from plasmid pMM1702 [69] that was modified to express fusion proteins containing both 10xHis- and FLAG-tags at their N-termini. Plasmid DNA was extracted from strain TH27367 using Zymo Research’s Plasmid Miniprep-Classic Kit following the provided protocol. The extracted plasmid DNA and purified PCR-generated *eGFP* DNA samples were digested with restriction enzymes NdeI and BamHI. Digested PCR-generated and plasmid DNA samples were then ligated together using T4 DNA ligase. The ligation reactions were electroporated into strain E*coli* XL1. The corresponding strains containing plasmids ptrc-99A-10His-FLAG-eGFP and ptrc-99A-10His-FLAG-eGFP(L201S) were isolated following Ap^R^ selection. The plasmid DNA sequences were verified, and purified plasmid DNA was introduced into *Salmonella* strains by electroporation.

### eGFP protein purification

The eGFP and eGFP(L201S)/ proteins were expressed from strain TH28567 (ptrc-His-Flag-*eGFP*/LT2) and strain TH28592 (ptrc-His-Flag-*eGFP*(L201S)/*hpaB*::Tn*10d*Tc *fla-5398* Δ*STM1941*::*cat leuZ*(A25G)). One liter of cells was grown at 37°C in LB supplemented with carbenicillin. At an OD_600_ of 0.6, IPTG was added to the growing cell culture at 0.25 mM. Cells were grown overnight and harvested the next day. Cells were centrifuged and the cell pellet was resuspended into 16 ml of lysis buffer (50 mM NaH_2_PO_4_; 300 mM NaCl; 10 mM imidazole). Lysozyme was added at a concentration of 1 mg/ml and the cells were sonicated (3 x 10 sec bursts with 15 sec cooling). The lysate was then centrifuged at 10,000 g for 30 mins at 4°C to pellet cellular debris. A 1ml portion of Ni-NTA resin (Qiagen) was added to the supernatant and the mixture was gently mixed at 4°C for 1 to 2 hrs. The slurry was then transferred onto a gravity column and the resin was washed with 4 ml of Wash Buffer (50 mM NaH_2_PO_4_; 300 mM NaCl; 20 mM imidazole). Bound protein was eluted in 0.5ml fractions of Elution buffer (50 mM NaH_2_PO_4_; 300 mM NaCl; 250 mM imidazole). Each fraction was analyzed by SDS-PAGE and the most concentrated eGFP fraction was sent to University of Arizona’s Analytical and Biological Mass Spectrometry Core for analysis.

### Intact protein analysis

LC-MS analyses of intact protein samples were analyzed by LC-MS using a Vanquish Horizon UHPLC Duo system (Thermo Scientific, San Jose, CA) coupled to an Orbitrap Exploris 480 mass spectrometer (Thermo Scientific, Bremen, Germany) fitted with an H-ESI source. Proteins were desalted and separated on a BioResolve RP mAb Polyphenyl Column (450Å, 2.7 µm, 2.1 mm X 50 mm, Waters Corp.) heated at 50°C with a gradient of 0.1% FA in ACN at 300 µl/min: from 10 to 90% in 15 min. For all experiments spray voltage was set to 3.8 kV, the sheath gas setting was 50, the auxiliary gas setting was 10, the sweep Gas 1 vaporizer temperature was 225°C, and the ion transfer tube temperature was 325°C. MS spectra were acquired at 7,500 resolving power (at m/z 200) with a scan range set to m/z 500- 4,000 Da, 10 microscans per MS scan, a normalized automatic gain control (AGC) target value of 300%, and a maximum injection time mode set to 200 ms. RF value was set to 60%. Source fragmentation energy on the Orbitrap Exploris 480 was 15V. MS spectra were deconvolved with BioPharma Finder 5.0 software.

### Nano-LC-MS/MS method and data searching

Nano-LC-MS/MS analysis was performed on a Q Exactive Plus mass spectrometer (Thermo Fisher Scientific, San Jose, CA) equipped with an EASY-Spray nanoESI source. The protein sample were digested with trypsin using micro spin columns using the manufacturer provided protocol. The resulted peptides (300 ng) were eluted from an Acclaim Pepmap 100 trap column (75 micron ID x 2 cm, Thermo Scientific) onto an Acclaim PepMap RSLC analytical column (75 micron ID × 25 cm, Thermo Scientific using a 3-25% gradient of solvent B (acetonitrile, 0.1% formic acid) over 150 min, 25-50% solvent B over 20 min, 50-70% of solvent B over 10 min, 70-95% B over 10 min then a hold of solvent 95% B for 20 min, and finally a return to 3% solvent B for 10 min. Solvent A consisted of water and 0.1% formic acid. Flow rates were 300 nL/min using a Vanquish Neo UHPLC System (Thermo Fisher Scientific, San Jose, CA). Data dependent scanning was performed by the Xcalibur v 4.3.73.11 software using a survey scan at 70,000 resolution scanning mass/charge (m/z) 300-1600 at an automatic gain control (AGC) target of 1e6 and a maximum injection time (IT) of 65 msec, followed by higher-energy collisional dissociation (HCD) tandem mass spectrometry (MS/MS) at 27 (N)CE (normalized collision energy), of the 11 most intense ions at a resolution of 17,500, an isolation width of 1.5 m/z, an AGC of 5e4 and a maximum IT of 65 msec. Dynamic exclusion was set to place any selected m/z on an exclusion list for 30 seconds after a single MS/MS. Ions of charge state +1, 6-8, >8, unassigned, and isotopes were excluded from MS/MS acquisition.

### Protein identification

MS and MS/MS data were searched against the amino acid sequence of the customized database using Thermo Proteome Discoverer v 2.4.1.15 (Thermo Fisher Scientific). MS/MS spectra matches considered fully tryptic peptides with up to 2 missed cleavage sites. Variable modifications considered were methylthio on Cys (+45.988Da) and Met (M) oxidation (+15.995Da). Proteins were identified at 95% confidence with XCorr score cut-offs as determined by a reversed database search.

### Translation inhibition to detect eGFP(L201S) fluorescence

Overnight cultures of strain TH29056 (ptrc-His-Flag-*eGFP*(L201S)**/** *leuZ*(A25G) *pnp*(I654T)), strain TH29058 (ptrc-His-Flag-*eGFP*(L201S)**/***argG*::Tn*10d*Cm *pnp*(I654T)), and strain TH29134 (ptrc-His-Flag-*eGFP*(L201S)/ *pnp*(I654T)) were grown at 30°C in LB supplemented with 100 μg/ml Ap and 250 μM IPTG. The overnight culture was diluted, 1:100, into fresh medium and grown until it reached an OD_600_ of 0.4. A 1 ml portion of cell culture was spun down and resuspended into 100 μl of buffered saline. Agarose pads were prepared on microscope slides for the detection of functional eGFP protein, which results in cell fluorescence. The agar pads contained 1% agarose supplemented with minimal E-salts medium containing 50μg/ml Ap, 250 μM IPTG, and 0.6 mM arginine. A 10μl portion of cells were spread onto the entire surface of each agarose pad. Liquid antibiotic solutions were applied onto each microscope slide at one edge yielding a final applied amount of antibiotic of 3μg for Tc, 10μg for Kasugamycin, 20μg for Spectinomycin, and 2.5μg for Cm. A coverslip was placed onto each agarose pad. The microscope slide was incubated at 37°C for 6 hours, then left at room temperature until the following day. Fluorescent microscopy analysis was performed using a Zeiss Axio Observer microscope equipped with a 100x oil immersion objective, a Solid-State Light Source Colibri 7 and an Axiocam 705 mono camera. Images were acquired and generated using the Zeiss Zen 3.7 software every 5-10 steps starting at the zone of inhibition. One step is equal to 0.89 μm. The average fluorescence per cell was determined by dividing the total fluorescence by the number of cells in the captured image. The average fluorescence/cell was then plotted on a graph against the distance from the Zone of Inhibition (expressed in steps). Each antibiotic was tested in triplicate for each strain. The average between each replicate was calculated using Microsoft Excel and plotted on the graph.

## Supporting information

S1 Table List of strains. (PDF)

S2 Table Lists of oligos. (PDF)

S3 Table β-galatosidase activity values for samples analyzed in Figs 5 and 6. *All strains carry the hisD9953::MudJ insertion. **These codons correspond to the His4 and His5 codon positions in the His leader peptide. ***Assays were performed 3 to 6 times from independent cultures. Activity is expressed in nmol/min/OD650.
(PDF)

S1 Fig Next generation sequence results for four, independent spontaneous Mac-Lac red colony revertants from strain TH27698 (hisL10592(His4-5 = TCA-TAC) hisD993::MudJ thrU(C40A)) as described in Methods. Suppressor 1 was subject to Nanopore Sequencing (Plasmidsaurus) and suppressors 2 through 4 were sequenced using Illumina Sequencing (University of Utah Core Facility) for comparative purposes.
(PDF)

## References

[1] Mustafi M, Weisshaar JC. Simultaneous binding of multiple EF-Tu copies to translating ribosomes in live *Escherichia coli*. mBio. 2018;9(1):e02143-17. doi: 10.1128/mBio.02143-17 29339430 PMC5770553

[2] Rudorf S, Thommen M, Rodnina MV, Lipowsky R. Deducing the kinetics of protein synthesis in vivo from the transition rates measured in vitro. PLoS Comput Biol. 2014;10(10):e1003909. doi: 10.1371/journal.pcbi.1003909 25358034 PMC4214572

[3] Morse JC, Girodat D, Burnett BJ, Holm M, Altman RB, Sanbonmatsu KY, et al. Elongation factor-Tu can repetitively engage aminoacyl-tRNA within the ribosome during the proofreading stage of tRNA selection. Proc Natl Acad Sci USA. 2020;117(7):3610–20. doi: 10.1073/pnas.1904469117 32024753 PMC7035488

[4] Diaconu M, Kothe U, Schlünzen F, Fischer N, Harms JM, Tonevitsky AG, et al. Structural basis for the function of the ribosomal L7/12 stalk in factor binding and GTPase activation. Cell. 2005;121(7):991–1004. doi: 10.1016/j.cell.2005.04.015 15989950

[5] Kothe U, Wieden H-J, Mohr D, Rodnina MV. Interaction of helix D of elongation factor Tu with helices 4 and 5 of protein L7/12 on the ribosome. J Mol Biol. 2004;336(5):1011–21. doi: 10.1016/j.jmb.2003.12.080 15037065

[6] Fischer N, Neumann P, Bock LV, Maracci C, Wang Z, Paleskava A, et al. The pathway to GTPase activation of elongation factor SelB on the ribosome. Nature. 2016;540(7631):80–5. doi: 10.1038/nature20560 27842381

[7] Loveland AB, Demo G, Grigorieff N, Korostelev AA. Ensemble cryo-EM elucidates the mechanism of translation fidelity. Nature. 2017;546(7656):113–7. doi: 10.1038/nature22397 28538735 PMC5657493

[8] Demeshkina N, Jenner L, Westhof E, Yusupov M, Yusupova G. A new understanding of the decoding principle on the ribosome. Nature. 2012;484(7393):256–9. doi: 10.1038/nature10913 22437501

[9] Ogle JM, Brodersen DE, Clemons WM Jr, Tarry MJ, Carter AP, Ramakrishnan V. Recognition of cognate transfer RNA by the 30S ribosomal subunit. Science. 2001;292(5518):897–902. doi: 10.1126/science.1060612 11340196

[10] Dale T, Fahlman RP, Olejniczak M, Uhlenbeck OC. Specificity of the ribosomal A site for aminoacyl-tRNAs. Nucleic Acids Res. 2009;37(4):1202–10. doi: 10.1093/nar/gkn1040 19129224 PMC2651786

[11] Åqvist J, Kamerlin SCL. The conformation of a catalytic loop is central to GTPase activity on the ribosome. Biochemistry. 2015;54(2):546–56. doi: 10.1021/bi501373g 25515218

[12] Wool IG, Glück A, Endo Y. Ribotoxin recognition of ribosomal RNA and a proposal for the mechanism of translocation. Trends Biochem Sci. 1992;17(7):266–9. doi: 10.1016/0968-0004(92)90407-z 1502728

[13] Schmeing TM, Voorhees RM, Kelley AC, Gao Y-G, Murphy FV 4th, Weir JR, et al. The crystal structure of the ribosome bound to EF-Tu and aminoacyl-tRNA. Science. 2009;326(5953):688–94. doi: 10.1126/science.1179700 19833920 PMC3763470

[14] Schuette J-C, Murphy FV 4th, Kelley AC, Weir JR, Giesebrecht J, Connell SR, et al. GTPase activation of elongation factor EF-Tu by the ribosome during decoding. EMBO J. 2009;28(6):755–65. doi: 10.1038/emboj.2009.26 19229291 PMC2666022

[15] Schmeing TM, Voorhees RM, Kelley AC, Ramakrishnan V. How mutations in tRNA distant from the anticodon affect the fidelity of decoding. Nat Struct Mol Biol. 2011;18(4):432–6. doi: 10.1038/nsmb.2003 21378964 PMC3072312

[16] Voorhees RM, Schmeing TM, Kelley AC, Ramakrishnan V. The mechanism for activation of GTP hydrolysis on the ribosome. Science. 2010;330(6005):835–8. doi: 10.1126/science.1194460 21051640 PMC3763471

[17] Maracci C, Peske F, Dannies E, Pohl C, Rodnina MV. Ribosome-induced tuning of GTP hydrolysis by a translational GTPase. Proc Natl Acad Sci USA. 2014;111(40):14418–23. doi: 10.1073/pnas.1412676111 25246550 PMC4210003

[18] Caulfield T, Devkota B. Motion of transfer RNA from the A/T state into the A-site using docking and simulations. Proteins. 2012;80(11):2489–500. doi: 10.1002/prot.24131 22730134

[19] Valle M, Zavialov A, Li W, Stagg SM, Sengupta J, Nielsen RC, et al. Incorporation of aminoacyl-tRNA into the ribosome as seen by cryo-electron microscopy. Nat Struct Biol. 2003;10(11):899–906. doi: 10.1038/nsb1003 14566331

[20] Mohr D, Wintermeyer W, Rodnina MV. GTPase activation of elongation factors Tu and G on the ribosome. Biochemistry. 2002;41(41):12520–8. doi: 10.1021/bi026301y 12369843

[21] Girodat D, Blanchard SC, Wieden H-J, Sanbonmatsu KY. Elongation factor Tu switch I element is a gate for aminoacyl-tRNA selection. J Mol Biol. 2020;432(9):3064–77. doi: 10.1016/j.jmb.2020.01.038 32061931 PMC8259901

[22] Frank J, Agrawal RK. A ratchet-like inter-subunit reorganization of the ribosome during translocation. Nature. 2000;406(6793):318–22. doi: 10.1038/35018597 10917535

[23] Presnyak V, Alhusaini N, Chen Y-H, Martin S, Morris N, Kline N, et al. Codon optimality is a major determinant of mRNA stability. Cell. 2015;160(6):1111–24. doi: 10.1016/j.cell.2015.02.029 25768907 PMC4359748

[24] Komar AA, Lesnik T, Reiss C. Synonymous codon substitutions affect ribosome traffic and protein folding during in vitro translation. FEBS Lett. 1999;462(3):387–91. doi: 10.1016/s0014-5793(99)01566-5 10622731

[25] Kudla G, Murray AW, Tollervey D, Plotkin JB. Coding-sequence determinants of gene expression in *Escherichia coli*. Science. 2009;324(5924):255–8. doi: 10.1126/science.1170160 19359587 PMC3902468

[26] Zhou M, Guo J, Cha J, Chae M, Chen S, Barral JM, et al. Non-optimal codon usage affects expression, structure and function of clock protein FRQ. Nature. 2013;495(7439):111–5. doi: 10.1038/nature11833 23417067 PMC3629845

[27] Ogle JM, Carter AP, Ramakrishnan V. Insights into the decoding mechanism from recent ribosome structures. Trends Biochem Sci. 2003;28(5):259–66. doi: 10.1016/S0968-0004(03)00066-5 12765838

[28] Gromadski KB, Daviter T, Rodnina MV. A uniform response to mismatches in codon-anticodon complexes ensures ribosomal fidelity. Mol Cell. 2006;21(3):369–77. doi: 10.1016/j.molcel.2005.12.018 16455492

[29] Rojiani MV, Jakubowski H, Goldman E. Relationship between protein synthesis and concentrations of charged and uncharged tRNATrp in *Escherichia coli*. Proc Natl Acad Sci USA. 1990;87(4):1511–5. doi: 10.1073/pnas.87.4.1511 2106136 PMC53505

[30] Elf J, Nilsson D, Tenson T, Ehrenberg M. Selective charging of tRNA isoacceptors explains patterns of codon usage. Science. 2003;300(5626):1718–22. doi: 10.1126/science.1083811 12805541

[31] Bossi L, Roth JR. The influence of codon context on genetic code translation. Nature. 1980;286(5769):123–7. doi: 10.1038/286123a0 7402305

[32] Bouadloun F, Srichaiyo T, Isaksson LA, Björk GR. Influence of modification next to the anticodon in tRNA on codon context sensitivity of translational suppression and accuracy. J Bacteriol. 1986;166(3):1022–7. doi: 10.1128/jb.166.3.1022-1027.1986 3086285 PMC215227

[33] Björnsson A, Isaksson LA. UGA codon context which spans three codons. Reversal by ms2i6A37 in tRNA, mutation in rpsD(S4) or streptomycin. J Mol Biol. 1993;232(4):1017–29. doi: 10.1006/jmbi.1993.1457 8371264

[34] Chevance FFV, Hughes KT. Case for the genetic code as a triplet of triplets. Proc Natl Acad Sci USA. 2017;114(18):4745–50. doi: 10.1073/pnas.1614896114 28416671 PMC5422812

[35] Johnston HM, Barnes WM, Chumley FG, Bossi L, Roth JR. Model for regulation of the histidine operon of Salmonella. Proc Natl Acad Sci USA. 1980;77(1):508–12. doi: 10.1073/pnas.77.1.508 6987654 PMC348301

[36] Johnston HM, Roth JR. Genetic analysis of the histidine operon control region of *Salmonella* typhimurium. J Mol Biol. 1981;145(4):713–34. doi: 10.1016/0022-2836(81)90311-9 7021855

[37] Johnston HM, Roth JR. DNA sequence changes of mutations altering attenuation control of the histidine operon of *Salmonella* typhimurium. J Mol Biol. 1981;145(4):735–56. doi: 10.1016/0022-2836(81)90312-0 6167727

[38] Spöring I, M V, Hotz C, Schwarz-Linek J, Grady KL, Nava-Sedeño JM, et al. Hlotbfiofmsotf bundle. PBS. 16(9):e2006989. 10.1371/journal.pbio.2006989 PMC6126814 30188886

[39] Chevance FFV, Le Guyon S, Hughes KT. The effects of codon context on in vivo translation speed. PLoS Genet. 2014;10(6):e1004392. doi: 10.1371/journal.pgen.1004392 24901308 PMC4046918

[40] Li X-T, Thomason LC, Sawitzke JA, Costantino N, Court DL. Positive and negative selection using the tetA-sacB cassette: recombineering and P1 transduction in *Escherichia coli*. Nucleic Acids Res. 2013;41(22):e204. doi: 10.1093/nar/gkt1075 24203710 PMC3905872

[41] Busby S, Ebright RH. Transcription activation by catabolite activator protein (CAP). J Mol Biol. 1999;293(2):199–213. doi: 10.1006/jmbi.1999.3161 10550204

[42] Gross CA, Chan C, Dombroski A, Gruber T, Sharp M, Tupy J, et al. The functional and regulatory roles of sigma factors in transcription. Cold Spring Harb Symp Quant Biol. 1998;63:141–55. doi: 10.1101/sqb.1998.63.141 10384278

[43] Pavlov MY, Watts RE, Tan Z, Cornish VW, Ehrenberg M, Forster AC. Slow peptide bond formation by proline and other N-alkylamino acids in translation. Proc Natl Acad Sci USA. 2009;106(1):50–4. doi: 10.1073/pnas.0809211106 19104062 PMC2629218

[44] Petrone PM, Snow CD, Lucent D, Pande VS. Side-chain recognition and gating in the ribosome exit tunnel. Proc Natl Acad Sci USA. 2008;105(43):16549–54. doi: 10.1073/pnas.0801795105 18946046 PMC2575457

[45] Chaconas G, de Bruijn FJ, Casadaban MJ, Lupski JR, Kwoh TJ, Harshey RM, et al. In vitro and in vivo manipulations of bacteriophage Mu DNA: cloning of Mu ends and construction of mini-Mu’s carrying selectable markers. Gene. 1981;13(1):37–46. doi: 10.1016/0378-1119(81)90041-x 6263754

[46] Anderson P, Roth J. Spontaneous tandem genetic duplications in *Salmonella* typhimurium arise by unequal recombination between rRNA (rrn) cistrons. Proc Natl Acad Sci USA. 1981;78(5):3113–7. doi: 10.1073/pnas.78.5.3113 6789329 PMC319510

[47] Aiso T, Ohki R. An rne-1 pnp-7 double mutation suppresses the temperature-sensitive defect of lacZ gene expression in a divE mutant. J Bacteriol. 1998;180(6):1389–95. doi: 10.1128/JB.180.6.1389-1395.1998 9515904 PMC107035

[48] Li Z, Reimers S, Pandit S, Deutscher MP. RNA quality control: degradation of defective transfer RNA. EMBO J. 2002;21(5):1132–8. doi: 10.1093/emboj/21.5.1132 11867541 PMC125898

[49] Shi Z, Yang W-Z, Lin-Chao S, Chak K-F, Yuan HS. Crystal structure of *Escherichia coli* PNPase: central channel residues are involved in processive RNA degradation. RNA. 2008;14(11):2361–71.doi: 10.1261/rna.1244308 18812438 PMC2578853

[50] Bezerra AR, Simões J, Lee W, Rung J, Weil T, Gut IG, et al. Reversion of a fungal genetic code alteration links proteome instability with genomic and phenotypic diversification. Proc Natl Acad Sci USA. 2013;110(27):11079–84. doi: 10.1073/pnas.1302094110 23776239 PMC3704024

[51] Tamura F, Nishimura S, Ohki M. The *E. coli* divE mutation, which differentially inhibits synthesis of certain proteins, is in tRNASer1. EMBO J. 1984;3(5):1103–7. doi: 10.1002/j.1460-2075.1984.tb01936.x 6376117 PMC557480

[52] Chevance FFV, Karlinsey JE, Wozniak CE, Hughes KT. A little gene with big effects: a serT mutant is defective in flgM gene translation. J Bacteriol. 2006;188(1):297–304. doi: 10.1128/JB.188.1.297-304.2006 16352846 PMC1317601

[53] Ude S, Lassak J, Starosta AL, Kraxenberger T, Wilson DN, Jung K. Translation elongation factor EF-P alleviates ribosome stalling at polyproline stretches. Science. 2013;339(6115):82–5. doi: 10.1126/science.1228985 23239623

[54] Wee LM, Tong AB, Florez Ariza AJ, Cañari-Chumpitaz C, Grob P, Nogales E, et al. A trailing ribosome speeds up RNA polymerase at the expense of transcript fidelity via force and allostery. Cell. 2023;186(6):1244–62.e34. doi: 10.1016/j.cell.2023.02.008 36931247 PMC10135430

[55] Burmann BM, Schweimer K, Luo X, Wahl MC, Stitt BL, Gottesman ME, et al. A NusE:NusG complex links transcription and translation. Science. 2010;328(5977):501–4. doi: 10.1126/science.1184953 20413501

[56] Arenz S, Wilson DN. Bacterial protein synthesis as a target for antibiotic inhibition. Cold Spring Harb Perspect Med. 2016;6(9):a025361. doi: 10.1101/cshperspect.a025361 27481773 PMC5008061

[57] Gromadski KB, Rodnina MV. Kinetic determinants of high-fidelity tRNA discrimination on the ribosome. Mol Cell. 2004;13(2):191–200. doi: 10.1016/s1097-2765(04)00005-x 14759365

[58] Cochella L, Green R. An active role for tRNA in decoding beyond codon:anticodon pairing. Science. 2005;308(5725):1178–80. doi: 10.1126/science.1111408 15905403 PMC1687177

[59] Hirsh D. Tryptophan transfer RNA as the UGA suppressor. J Mol Biol. 1971;58(2):439–58. doi: 10.1016/0022-2836(71)90362-7 4933412

[60] Bouadloun F, Donner D, Kurland CG. Codon-specific missense errors in vivo. EMBO J. 1983;2(8):1351–6. doi: 10.1002/j.1460-2075.1983.tb01591.x 10872330 PMC555282

[61] Edelmann P, Gallant J. Mistranslation in *E. coli*. Cell. 1977;10(1):131–7. doi: 10.1016/0092-8674(77)90147-7 138485

[62] Ruusala T, Andersson D, Ehrenberg M, Kurland CG. Hyper-accurate ribosomes inhibit growth. EMBO J. 1984;3(11):2575–80. doi: 10.1002/j.1460-2075.1984.tb02176.x 6391914 PMC557732

[63] Andersson DI, van Verseveld HW, Stouthamer AH, Kurland CG. Suboptimal growth with hyper-accurate ribosomes. Arch Microbiol. 1986;144(1):96–101. doi: 10.1007/BF00454963 3963992

[64] Karlinsey JE. lambda-red genetic engineering in *Salmonella enterica* serovar Typhimurium. Methods Enzymol. 2007;421:199–209. doi: 10.1016/S0076-6879(06)21016-4 17352924

[65] Davis RW, Botstein D, Roth JR. Advanced bacterial genetics. Cold Spring Harbor, N.Y.: Cold Spring Harbor Laboratory; 1980.

[66] Datsenko KA, Wanner BL. One-step inactivation of chromosomal genes in *Escherichia coli* K-12 using PCR products. Proc Natl Acad Sci USA. 2000;97(12):6640–5. doi: 10.1073/pnas.120163297 10829079 PMC18686

[67] Hughes KT, Roth JR. Directed formation of deletions and duplications using Mud(Ap, lac). Genetics. 1985;109(2):263–82. doi: 10.1093/genetics/109.2.263 3156064 PMC1202487

[68] Maloy SR. Experimental techniques in bacterial genetics. Boston: Jones and Bartlett; 1990.

[69] Minamino T, Macnab RM. Components of the *Salmonella flagellar* export apparatus and classification of export substrates. J Bacteriol. 1999;181(5):1388–94. doi: 10.1128/JB.181.5.1388-1394.1999 10049367 PMC93525

[70] Jenner LB, Demeshkina N, Yusupova G, Yusupov M. Structural aspects of messenger RNA reading frame maintenance by the ribosome. Nat Struct Mol Biol. 2010;17(5):555–60. doi: 10.1038/nsmb.1790 20400952

