# Supplementary material for: D-stem mutation in an essential tRNA increases translation speed at the cost of fidelity
Source: PLoS Genet. 2025 Feb 4;21(2):e1011569. doi: 10.1371/journal.pgen.1011569 (PMC11805395; doi:10.1371/journal.pgen.1011569)
Supplement: S1 Table — (PDF) [file pgen.1011569.s001.pdf]

S1 Table. List of Strains*

Strain #	Genotype	Source
TH85	Δ*his-3050*	John Roth
TH408	*nad*::Tn*10d*Tc	lab collection
TH437	Wild-type strain LT2	J. Roth
TH653	*hisD9953*::MudA	lab collection
TH2094	*fliN5103*::MudB	lab collection
TH2141	*hisD9953*::MudJ	[1]
TH4702	pKD46 Ap^{R}/LT2	[2]
TH5219	pKD3 (Cm^{R}) in *E. coli*	[2]
TH7012	*hpaB*::Tn*10d*Tc *fla-5398*(*serT*)	[3]
TH14175	*E. coli* XL1	lab collection
TH15419	Δ*hisG10504*::*eGFP*	lab collection
TH15759	*hisO10508*::*tetRA hisD9953*::MudJ	[4]
TH17685	*hisL10540*(His4-His5 = CCC-CCC) *hisD9953*::MudJ	[4]
TH23548	*E. coli Tet-sacB*	Don Court
TH24107	*hisL10555*::Km(before stop)	
TH24193	*hisO10508*::*tetRA*	
TH24401	*zdx-3729*::MudF	John Roth
TH24476	*argG*::Tn*10d*Cm Δ*araBAD2073*::*sipC-colA* Δ*prgH74* Δ*fliF7355* (Ara^{S})	
TH24352	*hisD10572*::*tetRA*	
TH24596	pKD46 (Ap^{R})/*hisD10572*::*tetRA*	
TH25421	Δ*truA*	
TH25692	pSIM6 (Ap^{R})/*hsdLT6 hsdSA29 hsdSB*	
TH26167	Δ*truA hisO10508*::*tetRA*	
TH27083	pSIM5/LT2	Don Court
TH27367	ptrc99A-His-flhA-CD1-Ap^{R}/MG1655	
TH27455	Δ*hisD10589*::sacB	
TH27456	pKD46 (Ap^{R})/Δ*hisD10589*::*sacB*	
TH27458	Δ*hisD10591*::(*sacB*-FCF**)	
TH27460	*hisL10555*::Km Δ*hisD10591*::(*sacB*-FCF)	
TH27461	*hisL10508*::*tetRA* Δ*hisD10591*::(*sacB*-FCF)	
TH27502	*hisL10508*::*tetRA* Δ*hisD10589*::*sacB*	
TH27503	DUP4147[(*hisD9953*)*MudA*(*fliN5103*)	
TH27504	DUP4147[(*hisD9953*)*MudA*(*fliN5103 his-3050*)]]	
TH27505	*hisL10592*(His4-His5= TCA-TAC) *hisD9953*::MudJ	
TH27506	DUP4148[(*hisL10592*(His4-His5= TCA-TAC) *hisD9953*)*MudJ*(*fliN5103 his-3050*)]	
TH27507	*hisL10592*(His4-His5= TCA-TAC) Δ*hisD10591*::(*sacB*-FCF)	

TH27508	DUP4148[(*hisL1059*2(His4-His5= TCA-TAC) *hisD9953*)*MudJ*(*fliN5103 hisL10592*(His4-His5= TCA-TAC) Δ*hisD10591*::(*sacB*-FCF))]
TH27509	DUP4148[(*hisL10592*(His4-His5= TCA-TAC) *hisD9953*)*MudJ*(*fliN5103 hisL10592*(His4-His5= TCA-TAC) Δ*hisD10591*::(*sacB*-FCF))] *thrU*(C40A)
TH27510	DUP4148[(*hisL10592*(His4-His5= TCA-TAC) *hisD9953*)*MudJ*(*fliN5103 hisL10592*(His4-His5= TCA-TAC) Δ*hisD10591*::(*sacB*-FCF))]) *leuZ*(A25G)
TH27511	DUP4148[(*hisL10592*(His4-His5= TCA-TAC) *hisD9953*)*MudJ*(*fliN5103 hisL10592*(His4-His5= TCA-TAC) Δ*hisD10591*::(*sacB*-FCF))] *rpoA*(E261K)
TH27512	DUP4148[(*hisL10592*(His4-His5= TCA-TAC) *hisD9953*)*MudJ*(*fliN5103 hisL10592*(His4-His5= TCA-TAC) Δ*hisD10591*::(*sacB*-FCF))] *rpoD*(R605L)
TH27686	*hisL10592*(His4-His5= TCA-TAC) Δ*hisD10591*::(*sacB*-FCF) *leuZ*(A25G)
TH27687	*hisL10592*(His4-His5= TCA-TAC) Δ*hisD10591*::(sacB-FCF) *thrU*(C40A)
TH27688	*hisL10508*::*tetRA leuZ*(A25G)
TH27689	*hisL10592*(His4-His5 = TCA-TAC) *hisD9953*::MudJ *leuZ*(A25G) *pnp*(I654T)
TH27690	pSIM5 Ap^R/*hisL10592*(His4-His5= TCA-TAC) *hisD9953*::MudJ *leuZ*(A25G)
TH27691	Δ*STM1941*::*tetRA leuZ*(A25G) *hisL10592*(His4-His5= TCA-TAC) *hisD9953*::MudJ
TH27693	Δ*STM1941*::*cat leuZ*(A25G) *hisL10592*(His4-His5= TCA-TAC) *hisD9953*::MudJ
TH27694	Δ*STM1941*::*tetRA leuZ*(A25G)
TH27696	Δ*STM1941*::*cat leuZ*(A25G)
TH27698	*hisL10592*(His4-His5= TCA-TAC) *hisD9953*::MudJ *thrU*(C40A)
TH27699	pSIM5 (Ap^R)/*hisL10592*(His4-His5= TCA-TAC) *hisD9953*::MudJ *thrU*(C40A)
TH27700	Δ*STM4140*::*tetRA thrU*(C40A) *hisL10592*(His4-His5= TCA-TAC) *hisD9953*::MudJ
TH28084	*hisL10598*(His4-His5= CGA-CGA) *hisD9953::*MudJ
TH28176	Δ*hisG10504*::*eGFP* Δ*truA*
TH28396	Δ*hisG10599*::*eGFP-tetRA*(ΔAA201) Δ*truA*
TH28397	Δ*hisG10600*::*eGFP*-(L201S(TCA)) Δ*truA*
TH28496	*leuZ*(A25G) *pnp*(I654T)
TH28497	*leuZ*(A25G) *pnp*(I654T) *hisL10540*(His4-His5= CCC-CCC) *hisD9953*::MudJ
TH28498	*leuZ*(A25G) *pnp*(I654T) *hisL10598*(His4-His5= CGA-CGA) *hisD9953*::MudJ

TH28501	*hisL10632*(His4-His5= TCA-AAT) *hisD9953*::MudJ *leuZ*(A25G) *pnp*(I654T)
TH28502	*hisL10633*(His4-His5= TCA-TTC) *hisD9953*::MudJ *leuZ*(A25G) *pnp*(I654T)
TH28503	*hisL10634*(His4-His5= TCA-TTT) *hisD9953*::MudJ *leuZ*(A25G) *pnp*(I654T)
TH28504	*hisL10635*(His4-His5= TCA-GCC) *hisD9953*::MudJ *leuZ*(A25G) *pnp*(I654T)
TH28505	*hisL10636*(His4-His5= GGC-TCA) *hisD9953*::MudJ *leuZ*(A25G) *pnp*(I654T)
TH28566	ptrc99A-[ATG-Gly-His10-SSGH1-ETK-His-Met(*NdeI*)-*eGFP*-Stop-*BamHI*] Ap^R/ *E. coli* XL1
TH28567	ptrc99a-[ATG-Gly-His10-SSGH1-ETK-His-Met(*NdeI*)-*eGFP*(L201S)-Stop-*BamHI*] Ap^R/*E. coli* XL1
TH28568	ptrc99a-eGFP Ap^R/LT2
TH28569	ptrc99a-eGFP(L201S) Ap^R/LT2
TH28570	ptrc99a-eGFP Ap^R/Δ*STM1941*::*cat leuZ*(A25G)
TH28571	ptrc99a-*eGFP*(L201S) Ap^R/Δ*STM1941*::*cat leuZ*(A25G)
TH28578	*argG*::Tn*10d*Cm *leuZ*(A25G) *pnp*(I654T)
TH28579	*argG*::Tn*10d*Cm Δ*STM1941*::*tetRA leuZ*(A25G)
TH28580	Δ*STM3278*::*tetRA*
TH28581	*argG*::Tn*10d*Cm *pnp*(I654T)
TH28583	*hpaB*::Tn*10d*Tc *fla-5398* (*serT*) Δ*STM1941*::*cat leuZ*(A25G)
TH28589	ptrc99a-*eGFP* Ap^R /*hpaB*::Tn*10d*Tc *fla-5389*(*serT*)
TH28590	ptrc99a-*eGFP*(L201S) Ap^R /*hpaB*::Tn*10d*Tc *fla-5389*(*serT*)
TH28591	ptrc99a-*eGFP* Ap^R /*hpaB*::Tn*10d*Tc *fla-5398* (serT) Δ*STM1941*::*cat leuZ*(A25G)
TH28592	ptrc99- *eGFP*(L201S) (Ap^R)/*hpaB*::Tn*10d*Tc *fla-5398*(*serT*) Δ*STM1941*::*cat leuZ*(A25G)
TH28679	*hisL10602*(His4-His5= CGU-CGU) Δ*hisD10589*::*sacB*
TH28680	*hisL10603*(His4-His5= CGU-CGG) Δ*hisD10589*::*sacB*
TH28681	*hisL10604*(His4-His5= CGU-AGA) Δ*hisD10589*::*sacB*
TH28682	*hisL10605*(His4-His5= CGG-AGA) Δ*hisD10589*::*sacB*
TH28683	*hisL10606*(His4-His5= CGU-AGG) Δ*hisD10589*::*sacB*
TH28684	*hisL10607*(His4-His5= CGA-AGG) Δ*hisD10589*::*sacB*
TH28790	*hisL10592*(His4-His5= TCA-TAC) Δ*hisD10608*::(*sacB-tetRA*(ΔAA259-469)-FCF)
TH28791	*hisL10592*(His4-His5= TCA-TAC) Δ*hisD10609*::(*sacB*(259CTG, 297CTC, 308CTA, 384CTC, 398CTC, 469CTG)-FCF)
TH28792	*hisL10592*(His4-His5= TCA-TAC) Δ*hisD10610*::(*sacB-tetRA*(ΔAA13-177 of *sacB,* 259CTG, 297CTC, 308CTA, 384CTC, 398CTC, 469CTG)-FCF)

TH28793	*hisL10592*(His4-His5= TCA-TAC) Δ*hisD10611*::(*sacB*(13CTC, 90CTG, 109CTG, 177CTG, 259CTG, 297CTC, 308CTA, 384CTC, 398CTC, 469CTG)-*tetRA*-FCF)	
TH28794	Δ*STM1941*::*tetRA leuZ*(A25G) *hisL10592*(His4-His5 = TCA-TAC) Δ*hisD10591*::(*sacB*-FCF)	
TH28796	Δ*STM4140*::*tetRA thrU*(C40A) *zdx-3729*::MudF	
TH28797	Δ*STM4140*::*tetRA thrU*(C40A) *hisD9953*::MudJ	
TH28798	Δ*STM4140*::*tetRA thrU*(C40A) *hisL10540*(His4-His5= CCC-CCC) *hisD9953*::MudJ	
TH28799	Δ*STM4140*::*tetRA thrU*(C40A) *hisL10598*(His4-His5= CGA-CGA) *hisD9953*::MudJ	
TH28864	Δ*STM1941*::*tetRA leuZ*(A25G) *hisL10592*(His4-His5= TCA-TAC) Δ*hisD10611*::(sacB(13CTC, 90CTG, 109CTG, 177CTG, 259CTG, 297CTC, 308CTA, 384CTC, 398CTC, 469CTG)-FCF)	
TH28865	*hisL10634*(His4-His5= TCA-TTT) *hisD9953*::MudJ	[4]
TH28866	*hisL10633*(His4-His5= TCA-TTC) *hisD9953*::MudJ	[4]
TH28867	*hisL10632*(His4-His5= TCA-AAT) *hisD9953*::MudJ	[4]
TH28868	*hisL10635*(His4-His5= TCA-GCC) *hisD9953*::MudJ	[4]
TH28869	*hisL10636*(His4-His5= GGC-TCA) *hisD9953*::MudJ	[4]
TH28870	*hisL10602*(His4-His5= CGU-CGU) *hisD9953*::MudJ	[4]
TH28871	*hisL10603*(His4-His5= CGU-CGG) *hisD9953*::MudJ	[4]
TH28872	*hisL10604*(His4-His5= CGU-AGA) *hisD9953*::MudJ	[4]
TH28873	*hisL10605*(His4-His5= CGG-AGA) *hisD9953*::MudJ	[4]
TH28874	*hisL10606*(His4-His5= CGU-AGG) *hisD9953*::MudJ	[4]
TH28875	*hisL10606*(His4-His5= CGA-AGG) *hisD9953*::MudJ	[4]
TH28890	*hisD9953*::MudJ *leuZ*(A25G) *pnp*(I654T)	
TH29056	ptrc99- *eGFP*(L201S) (Ap^{R})/ *leuZ*(A25G) *pnp*(I654T)	
TH29058	ptrc99- *eGFP*(L201S) (Ap^{R})/ *argG*::Tn*10d*Cm *pnp*(I654T)	
TH29133	*pnp*(I654T)	
TH29134	ptrc99- *eGFP*(L201S) (Ap^{R})/ *pnp*(I654T)	
TH29792	*hisL10643* (AA2=TGT(Cys) His4-His5= TCA-TAC) *hisD9953*::MudJ	
TH29793	*hisL10644* (AA2=GAC(Asp) His4-His5= TCA-TAC) *hisD9953*::MudJ	
TH29794	*hisL10645* (AA2=ACG(Thr) His4-His5= TCA-TAC) *hisD9953*::MudJ	
TH29795	*hisL10646* (AA2=ACT(Thr) His4-His5= TCA-TAC) *hisD9953*::MudJ	
TH29796	*hisL10647* (AA2=GTT(Val) His4-His5= TCA-TAC) *hisD9953*::MudJ	
TH29797	*hisL10643* (AA2=TGT(Cys) His4-His5= TCA-TAC) *hisD9953*::MudJ *thrU*(C40A)	

TH29798	*hisL10644* (AA2=GAC(Asp) His4-His5= TCA-TAC) *hisD9953*::MudJ *thrU*(C40A)
TH29799	*hisL10645* (AA2=ACG(Thr) His4-His5= TCA-TAC) *hisD9953*::MudJ *thrU*(C40A)
TH29800	*hisL10646* (AA2=ACT(Thr) His4-His5= TCA-TAC) *hisD9953*::MudJ *thrU*(C40A)
TH29801	*hisL10647* (AA2=GTT(Val) His4-His5= TCA-TAC) *hisD9953*::MudJ *thrU*(C40A)

*Unless indicated otherwise, all strains were constructed during the course of this work
**FCF is the FRT-Cm^R-FRT cassette described in [2].

References

1. Hughes KT, Roth JR. Transitory *cis* complementation: a method for providing transposition functions to defective transposons. Genetics. 1988;119(1):9-12. PubMed PMID: 2840333.

2. Datsenko KA, Wanner BL. One-step inactivation of chromosomal genes in *Escherichia coli* K-12 using PCR products. Proc Natl Acad Sci U S A. 2000;97(12):6640-5. Epub 2000/06/01. doi: 10.1073/pnas.120163297 [pii]. PubMed PMID: 10829079.

3. Chevance FF, Karlinsey JE, Wozniak CE, Hughes KT. A little gene with big effects: a *serT* mutant is defective in *flgM* gene translation. J Bacteriol. 2006;188(1):297-304. doi: 188/1/297 [pii] 10.1128/JB.188.1.297-304.2006. PubMed PMID: 16352846.

4. Chevance FF, Le Guyon S, Hughes KT. The effects of codon context on *in vivo* translation speed. PLoS Genetics. 2014;10(6):e1004392. doi: 10.1371/journal.pgen.1004392. PubMed PMID: 24901308; PubMed Central PMCID: PMC4046918.
