# Supplementary material for: D-stem mutation in an essential tRNA increases translation speed at the cost of fidelity
Source: PLoS Genet. 2025 Feb 4;21(2):e1011569. doi: 10.1371/journal.pgen.1011569 (PMC11805395; doi:10.1371/journal.pgen.1011569)
Supplement: S2 Table — (PDF) [file pgen.1011569.s002.pdf]

S2 Table. List of Oligonucleotides

Primer Number and Name	DNA sequence (5'-3')
7999-hisleader-fw	caaatgaataagcattcatcggaatttttATGacacgcgttcaatttaaacacca
8000-hisleader-rv	atgcaccactggaagatctgaatgtcttccagcacacatcgcctgaaagacta
8094-His3NNN-Ser-Tyr Rev	gaagatctgaatgtcttccagcacacatcgcctgaaagactagtcaggatgatggtatga
8125-his345-NNN-Ser-tyr	cgcgttcaatttaaacaccacNNNTCATACcatcatcctgacTAGtctttcagg
8256-His adapter fw	acactctttccctacacgacgctcttccgatctgcgcttgctttaaggcgt
8257-his-adapter-rev	gactggagttcagacgtgtgctcttccgatct atgcgttcatgcaccactg
8269- HisDFRT-For	gcggcgcaacaccggatttcgtcgcttctgacctgctctcgtgtaggctggagctgcttc
8270- HisDFRT-Rev	cagcaggatcacctgggaatccgggccgtgctcagcctggcatatgaatatcctccttag
8277- HisDSacB-F	taggccggataaggcggaaccctgtgatggagtaaagaccatgaacatcaaaaagtttgc
8278- HisDSacB-R	tcgcctgcgcgccgccgacgttaaagatttcctgcctatttgttaactgttaattgtcct
8484-His2NNNSerTyr	acacgcgttcaatttaaacacNNNcattcataccatcatcctgacTAGtctttcaggcgatgtg
8896- STM4140tetR	gctttcacagcgagattttttcttattattcctccccatct ttaagacccactttcacatt
8897- stm4140-tetA	cacatttaagttaatttggacaatgggcaggagggtaacaccctaagcacttgtctcctg
9496- SacB(AA13) tetR	acgatgaacatcaaaaagtttgcaaaacaagcaacagtattaagacccactttcacatt
9497- SacB(AA177) tetA	ttgtttgccgtaatgtttaccggagaaatcagtgtagaactaagcacttgtctcctg
9479- SacB(Leu13) For	acgatgaacatcaaaaagtttgcaaaacaagcaacagtactcacctttactaccgcactgctggc
9480- SacB(Leu90) Rev	gtagccgtgatagtttgcgacagtgccgtcagcgttttgcagtggccagctgtcccaaac
9481- SacB(Leu109) For	actgtcgcaaactatcacggctaccacatcgtctttgcactggccggagatcctaaaaat
9482- SacB(Leu177) Rev	ttgtttgccgtaatgtttaccggagaaatcagtgtagaacagacggatttttccgtcaga
9483- SacB(259) tetR	agagatcctcactacgtagaagataaaggccacaaatacttaagacccactttcacatt
9484- SacB(469) tetA	ccgccgacgttaaagatttcctgcctatttgttaactgtctaagcacttgtctcctg
9485- SacB(Leu259) For	agagatcctcactacgtagaagataaaggccacaaatacctggtatttgaagcaaacact
9486- SacB(Leu277) Rev	aagaatgatgtgcttttgccatagtatgctttgttaaagagagattcttcgccttggta
9487- SacB(Leu259) for2	agagatcctcactacgta
9488- SacB(277-308) Rev	agccgtgcgtttttttatcgctttgcagaagtttttgactttcttgacggaagaatgatgtgcttttg
9489- SacB(Leu308) For	gcaaagcgataaaaaaacgcacggctgagctagcaaacggcgctctcggtatgattgagct
9490- SacB(Leu384) Rev	cacaaggccagttttgttcagcggcttgtatgggccagtgagagaattagaaacataacc
9491- SacB(Leu398) For	ctcactggcccatacaagccgctgaacaaaactggccttgtgctcaaaatggatcttgatcct
9492- SacB(Leu469) Rev	ccgccgacgttaaagatttcctgcctatttgttaactgtcagttgtccttgttcaagga
9493- SacB seq Rev	atcgatgaactgctgtac
9494- SacB int For	catcaacggtgtagagga
9519- hisD-sacB-rev	ccgccgacgttaaagatt
9523- dSTM1941 tetR	gggtaaatcagccgataaaatccattaatctctataaaaattaagacccactttcacatt
9524- dSTM1941 tetA	attaaactgtgcagaaaacaccctgaacagttatatactcgtctaagcacttgtctcctg
9693- STM1941catFor	gggtaaatcagccgataaaatccattaatctctataaaaatcctggtgtccctgttgat
9694- STM1941catRev	ttaaactgtgcagaaaacaccctgaacagttatatactcgtctaacttacgccccgccctgcca
9827- hisL2NNN	caaatgaataagcattcatcggaatttttatgnnncgcgttcaatttaaacacca

Primer Number and Name	DNA sequence (5’-3’)
9971- dSTM3278 tetR	gggatatgaataaatcgacttattaccagagaaaatcatcttaagacccactttcacatt
9972- dSTM3278 tetA	tcggcgagtgcttccccgtgttcatcgaaaaaagcatcggcgctaagcacttgtctcctg
10005- QC eGFP (L201S) F	ctgcccgacaaccactactcaagcacccagtccgccctgagcaaa
10066- eGFP(dAA201) tetR	catcggcgacggccccgtgctgctgcccgacaaccactacttaagacccactttcacatt
10067- eGFP (dAA201) tetA	gcgcttctcgttggggtctttgctcagggcggactgggtgctctaagcacttgtctcctg
10068- eGFP fillin F	catcggcgacggccccgtgctgctgcccgacaaccactac
10069- eGFP fillin R	gtgatcgcgcttctcgttggggtctttgctcagggcggactg
10149- ndeI-eGFP-fw	aatgcgtagcatatgagcaaaggcgaagaac
10150- bamHI-eGFP-rv	ggctattataggatcctcatttatacagttcatccatgccga
10331-hisLdAA2tetR	aaaaggtatcaaatgaataagcattcatcggaatttttatgttaagacccactttcacatt
10858-Hisleader Thr2-His4-5 CAG-GAT-tetA	gtcaggatgatgGTATGAatggtggtgtttaaattgaacgcgCTAAGCACTTGTCCCTG
10859-hisL-2NNN-his4-5tca-tac fill	gtcaggatgatgGTATGAatggtggtgtttaaattgaacgcg
10864-HisLeader-fill-fw	agtggtttaggttaaaaggtatcaaatgaataagcattcatcgg
