# Supplementary material for: D-stem mutation in an essential tRNA increases translation speed at the cost of fidelity
Source: PLoS Genet. 2025 Feb 4;21(2):e1011569. doi: 10.1371/journal.pgen.1011569 (PMC11805395; doi:10.1371/journal.pgen.1011569)
Supplement: S3 Table — *All strains carry the hisD9953::MudJ insertion. **These codons correspond to the His4 and His5 codon positions in the His leader peptide. ***Assays were performed 3 to 6 times from independent cultures. Activity is expressed in nmol/min/OD650. (PDF) [file pgen.1011569.s003.pdf]

Strain Number	Relevant Genotype*	β-Gal Activity***
TH2141	Wild Type	8.1 ± 0.9
TH28797	*thrU*(C40A)	4.2 ± 0.2
TH27505	His4-5::TCA-TAC**	140 ± 13
TH27698	His4-5::TCA-TAC *thrU*(C40A)	44 ± 15
TH17685	His4-5::CCC-CCC	290 ± 8.9
TH28798	His4-5::CCC-CCC *thrU*(C40A)	49 ± 3.2
TH28084	His4-5::CGA-CGA	270 ± 17
TH28799	His4-5::CGA-CGA *thrU*(C40A)	49 ± 3.2
TH29795	hisLAA2::*thr*(ACT) His4-5::TCA-TAC	130 ± 4.3
TH29800	hisLAA2::*thr*(ACT) His4-5::TCA-TAC *thrU*(C40A)	40 ± 1.6
TH29794	hisLAA2::*thr*(ACG) His4-5::TCA-TAC	130 ± 4.6
TH29799	hisLAA2::*thr*(ACG) His4-5::TCA-TAC *thrU*(C40A)	40 ± 0.5
TH29792	hisLAA2::cys(TGT) His4-5::TCA-TAC	82 ± 2.5
TH29797	hisLAA2::cys(TGT) His4-5::TCA-TAC *thrU*(C40A)	19 ± 1.8
TH29793	hisLAA2::*asp*(GAC) His4-5::TCA-TAC	85 ± 3.1
TH29798	hisLAA2::*asp*(GAC) His4-5::TCA-TAC *thrU*(C40A)	21 ± 1.3
TH29796	hisLAA2:: *val*(GTT) His4-5::TCA-TAC	110 ± 7.4
TH29801	hisLAA2::*val*(GTT) His4-5::TCA-TAC *thrU*(C40A)	43 ± 3.3
TH24401	MudF	230 ± 2.2
TH28796	MudF *thrU*(C40A)	68 ± 11
TH28890	*leuZ*(C40A) *pnp*(I654T)	7.1 ± 0.7
TH27689	His4-5::TCA-TAC *leuZ*(C40A) *pnp*(I654T)	71 ± 4.2
TH28497	His4-5:: CCC-CCC *leuZ*(C40A) *pnp*(I654T)	290 ± 25
TH28497	His4-5::CGA-CGA *leuZ*(C40A) *pnp*(I654T)	270 ± 19
TH28865	His4-5::TCA-TTT	25 ± 0.9
TH28503	His4-5::TCA-TTT *leuZ*(C40A) *pnp*(I654T)	15 ± 0.7
TH28866	His4-5::TCA-TTC	25 ± 1.7

TH28502	His4-5::TCA-TTC *leuZ*(C40A) *pnp*(I654T)	16 ± 0.8
TH28867	His4-5::TCA-AAT	15 ± 0.6
TH28501	His4-5::UCA-AAU *leuZ*(C40A) *pnp*(I654T)	8 ± 0.4
TH28868	His4-5::TCA-GCC	17 ± 1.9
TH28504	His4-5::TCA-GCC *leuZ*(C40A) *pnp*(I654T)	8 ± 0.7
TH28869	His4-5::GGC-TCA	75 ± 6.5
TH28505	His4-5::GGC-TCA *leuZ*(C40A) *pnp*(I654T)	48 ± 2.3

S3 Table. β-galatosidase activity values for samples analyzed in Fig 5 and Fig 6. *All strains carry the *hisD9953*::MudJ insertion. **These codons correspond to the His4 and His5 codon positions in the His leader peptide. ***Assays were performed 3 to 6 times from independent cultures. Activity is expressed in nmol/min/OD650.
