# Supplementary material for: D-stem mutation in an essential tRNA increases translation speed at the cost of fidelity
Source: PLoS Genet. 2025 Feb 4;21(2):e1011569. doi: 10.1371/journal.pgen.1011569 (PMC11805395; doi:10.1371/journal.pgen.1011569)
Supplement: S1 Fig — Suppressor 1 was subject to Nanopore Sequencing (Plasmidsaurus) and suppressors 2 through 4 were sequenced using Illumina Sequencing (University of Utah Core Facility) for comparative purposes. (PDF) [file pgen.1011569.s004.pdf]

S1 Fig. Next generation sequence results for four, independent spontaneous Mac-Lac red colony revertants from strain TH27698 (*hisL10592*(His4-5 = TCA-TAC) *hisD993*::MudJ *thrU*(C40A)) as described in Methods. Suppressor 1 was subject to Nanopore Sequencing (Plasmidsaurus) and suppressors 2 through 4 were sequenced using Illumina Sequencing (University of Utah Core Facility) for comparative purposes.

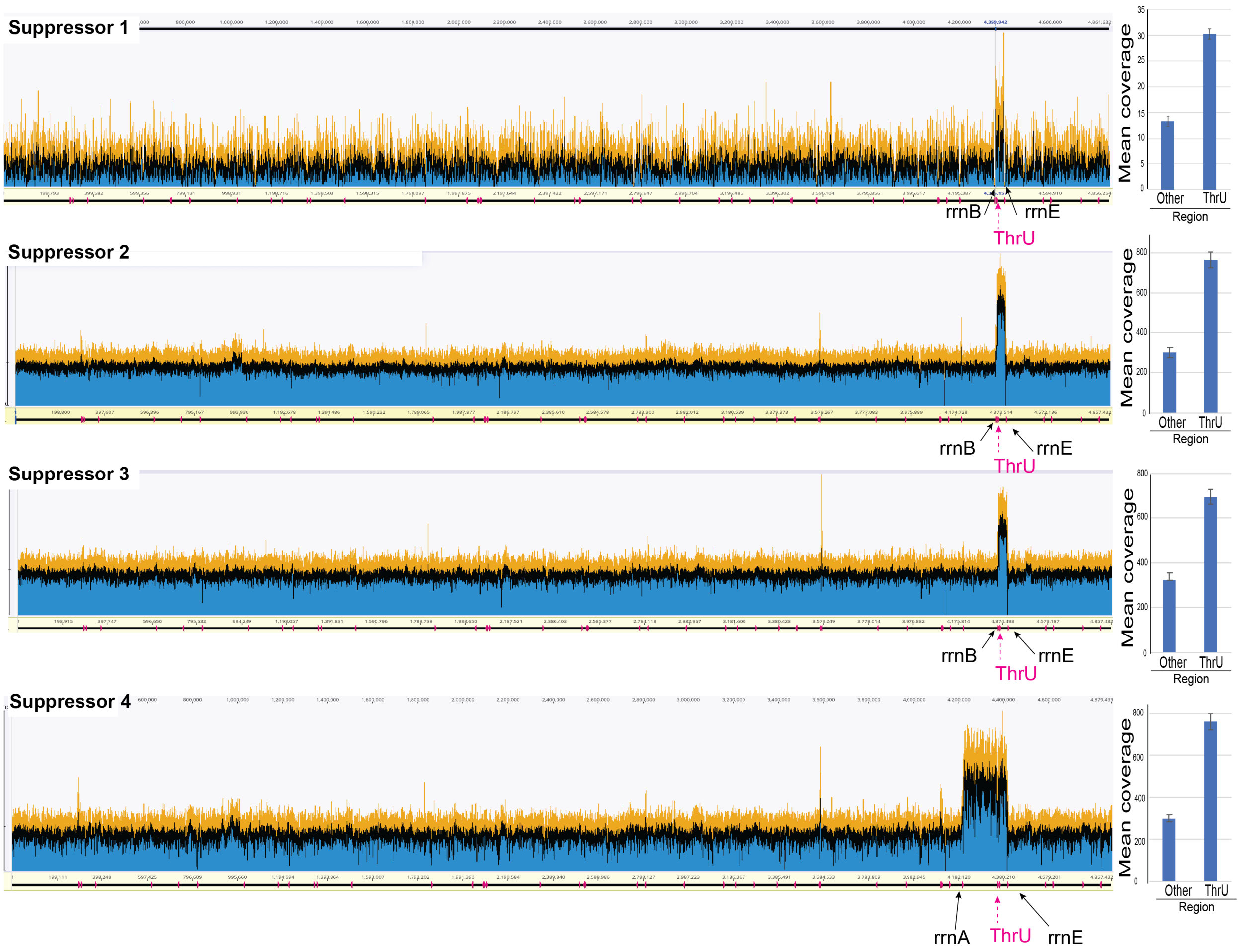
Suppressor 1
Mean coverage
Other
ThrU
Region
rrnB
rrnE
ThrU
Suppressor 2
Mean coverage
Other
ThrU
Region
rrnB
rrnE
ThrU
Suppressor 3
Mean coverage
Other
ThrU
Region
rrnB
rrnE
ThrU
Suppressor 4
Mean coverage
Other
ThrU
Region
rrnA
ThrU
rrnE

S1 Fig.
